# Diferrocenyl Thioketone: Reactions with (Bisphosphane)Pt(0) Complexes—Electrochemical and Computational Studies

**DOI:** 10.3390/ma12172832

**Published:** 2019-09-03

**Authors:** Sebastian Gröber, Piotr Matczak, Sławomir Domagała, Thomas Weisheit, Helmar Görls, Annika Düver, Grzegorz Mlostoń, Wolfgang Weigand

**Affiliations:** 1 Institute for Inorganic and Analytical Chemistry, Friedrich Schiller University Jena, Humboldstrasse 8, 07743 Jena, Germany; 2 Clinic of Nuclear Medicine, University Hospital Jena, Am Klinikum 1, 07747 Jena, Germany; 3 Department of Physical Chemistry, Faculty of Chemistry, University of Łódź, Pomorska 163/165, 90236 Lodz, Poland; 4 Department of Inorganic and Analytical Chemistry, Faculty of Chemistry, University of Łódź, Tamka 12, 91403 Łódź, Poland; 5 Department of Organic and Applied Chemistry, Faculty of Chemistry, University of Łódź, Tamka 12, 91403 Łódź, Poland

**Keywords:** thioketones, ferrocene, Pt(0) complexes, platinathiiranes, cyclic voltammetry, DFT calculations

## Abstract

Diferrocenyl thioketone reacts smoothly with (bisphosphane)Pt(0) complexes in toluene solution at room temperature yielding 1:1 adducts identified as ferrocenyl (Fc) functionalized platinathiiranes. Their structures were unambiguously confirmed by means of spectroscopic methods as well as by X-ray diffraction analysis. A unique, ferrocene-rich platinathiirane, bearing three Fc-units, was prepared starting with [bis(diphenylphosphino)ferrocene] Pt(0(*η*^2^-norbornene). For comparison, a similar platinathiirane with one Fc-unit was obtained from the reaction of the latter complex with thiobenzophenone. Quantum-chemical calculations were carried out to describe the bonding pattern and frontier molecular orbitals of the ferrocene-rich platinathiirane complexes. These calculations confirmed that the C=S bond loses its formally double-bond character upon complexation (bisphosphane)Pt(0). Cyclic voltammetry measurements were performed to characterize the obtained platinathiiranes in CH_2_Cl_2_ solutions. For comparison, the cyclic voltammogram for diferrocenyl thioketoneas a mixed-valent (Fe^II^-Fe^III^) compound was also recorded and analyzed. The results point out to a diffusion controlled electrode process in case of differocenyl thioketone and mixed diffusion and adsorption controlled electrode process in the case of the studied platinathiiranes.

## 1. Introduction

**Scheme 1:**
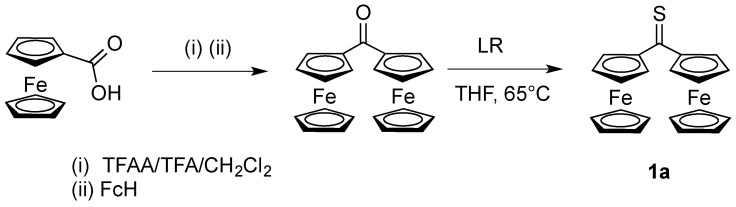
Preparation of thioketone **1a** by thionation of diferrocenyl ketone using Lawesson’s reagent (LR) [9]. TFAA/TFA/CH_2_Cl_2_ denotes trifluoroacetic anhydride in the presence of trifluoroacetic acid in solution.

In our continuing studies on the chemistry of thiocarbonyl compounds, we demonstrated that in contrast to information available in some earlier monographs [1], reviews [2], and textbooks [3], aryl, hetaryl, and ferrocenyl thioketones should currently be considered as versatile starting materials for the synthesis of more complex sulfur-containing as well as sulfur-free organic compounds [4]. In this context, their 1,3-dipolar cycloadditions and hetero-Diels-Alder reactions leading to five- or six-membered sulfur heterocycles, correspondingly, are of special importance. Because of the high reactivity in these reactions, thioketones were named as superdipolarophiles [5] and superdienophiles [6], respectively. It is well established that stability of thioketones and consequently their synthetic utility depend on the substitution pattern. Thus, non-enolisable cycloaliphatic thioketones as well as aryl- and hetaryl representatives can be prepared and used for further reactions with no special precautions [4]. In recent studies, we found that little known ferrocenyl thioketones are even more stable than their aryl analogues. For example, in contrast to the unstable thioacetophenone [7], ferrocenyl methyl thioketone is perfectly stable under standard conditions [8]. Similarly, diferrocenyl thioketone (**1a**) is also a stable compound, which can be easily prepared by thionation of diferrocenyl ketone using Lawesson´s reagent [9]. Elaboration of an efficient procedure for the preparation of diferrocenyl ketone and another ferrocenyl substituted ketones, was a challenging problem; in contrast to earlier procedures based on the Friedel–Crafts reaction with AlCl_3_ as a catalyst and acyl chlorides as acylating reagents [10,11], the protocol described in our recent publication, involving acylation of ferrocene with the in situ generated “mixed anhydride” is a method of choice to get to differocenyl ketone and related aryl/ferrocenyl as well hetaryl/ferrocenyl analogues in high, well reproducible yields [9] (Scheme 1).

In spite of the fact, that ferrocene itself is named as an exceptional molecule [12] and organic compounds functionalized with the electroactive ferrocenyl moiety are of great importance in diverse field of applied chemistry (materials chemistry, medicinal chemistry, electrochemistry), thioketone **1a** has been little explored in organic and coordination chemistry as a useful substrate for synthesis of such compounds and only few reports are known regarding data on its reactivity, e.g., thia-Diels-Alder reaction with 2,3-dimethyl-buta-1,3-diene yielded the corresponding 3,6-dihydro-2*H*-thiapyran [13] and the reaction with diphenyl diazomethane led after two-fold elimination of nitrogen [N_2_] and sulfur [S] to 2,2-diferrocenyl 3,3-diphenylthiirane [9]. In earlier reports, M(CO)_5_ complexes (M = Cr, W) of **1a** were obtained and their structures were studied by means of spectroscopic and X-ray diffraction (XRD) methods [14,15]. These studies showed that the M(CO)_5_ moiety is coordinated via the sulfur atom of the C=S group.

**Figure 1:**
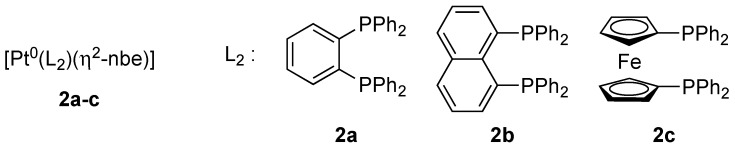
Bidentate phosphanes used as ligands in platinum complexes **2a**–**c**. L_2_ = 1,2-bis(diphenylphosphino)benzene (dppbe), 1,8-bis(diphenylphosphino)naphthalene (dppn), 1,1’-bis(diphenylphosphino)ferrocene (dppf); nbe = norbornene.

In a series of our recent publications we described the formation of platinathiiranes **3** in reactions of [Pt(0)L_2_(*η*^2^-olefine)] (L = phosphanes) **2** (Figure 1) with diverse aromatic and cycloaliphatic thioketones [16]. 

The same products were also obtained by treatment of 1,2,4-trithiolanes with **2.** In the case of dispiro-cycloaliphatic substituted 1,2,4-trithiolanes, the initiating step is the oxidative insertion of the L_2_Pt(0) complex fragment into the sulfur–sulfur bond of the 1,2,4-trithiolane ring. Subsequent (3 + 2)-cycloelimination of the intermediate formed thereby, releases the corresponding thioketone which in the next step interacts with another equivalent of **2** forming platinathiiranes **3**. In these multi-step reactions, the second type of products formed, was identified as four membered (dithiolato)platinum(II) complexes **4** [17].

**Scheme 2:**
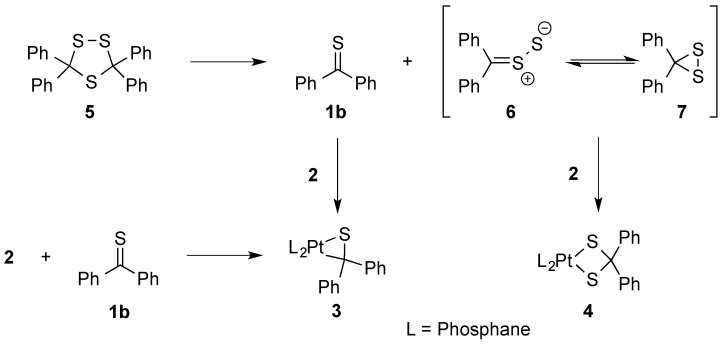
Preparation of platinathiiranes **3** and dithiolato complexes **4** in reactions with 1,2,4-trithiolane **5**.

On the other hand, in reactions of 3,3,5,5-tetraphenyl 1,2,4-trithiolane (**5**) another mechanism comprising the in situ generated diphenyldithiirane/thiobenzophenonen-S-sulfide (**6**/**7**) and thiobenzophenone (**1b**), which are both formed upon thermal (3 + 2)-cycloreversion of **5**, was postulated. These components are trapped with a phosphine complex of type **2** yielding side by side the corresponding platinathiiranes **3** and dithiolato complexes **4**, respectively. Complex **3** was also formed by direct reaction of **2** with **1b** (Scheme 2) [18].

Reactions of 1,2,4-trithiolanes and their *S*-oxides with Pt(0) complexes leading to diverse platinathiiranes and dithiolato complexes of type **3** and **4**, respectively, are summarized in a recent review [19].

The goal of the present work is the synthesis and characterization of novel platinathiiranes of type **3** derived from thioketone **1a** and diverse (bisphosphane)Pt(0) complexes of type **2** presented in Figure 1. Furthermore, physico-chemical properties and structures of the ferrocenyl functionalized platinathiiranes **3** should be studied by means of electrochemical and computational methods.

## 2. Materials and Methods

### 2.1. Preparation and Identification of Platinathiiranes ***3a**–**d***

*Materials and Techniques*. The ^1^H and ^13^C{^1^H} spectra were recorded with a Bruker Avance 200 MHz spectrometer (Terre Haute, IN, USA). Chemical shifts are given in parts per million with reference to internal SiMe_4_ or CHCl_3_. The mass spectrum was recorded with a Finnigan MAT SSQ 710 instrument (Auburn, CA, USA). The IR spectra were measured with a Perkin–Elmer System 2000 FT-IR spectrometer (Waltham, MA, USA). Elemental analysis was performed with a Leco CHNS-932 apparatus (Madrid, Spain). Silica gel 60 (0.015–0.040 mm) was used for column chromatography, and thin layer chromatography (TLC) was performed by using Merck (Kenilworth, NJ, USA) TLC aluminum sheets (Silica gel 60 F254). Solvents from Fisher Scientific (Ottawa, Ontario, Canada) and other chemicals from Acros were used without further purification. All solvents were dried and distilled prior to the use according to standard methods. The starting materials were prepared according to literature procedures [9,18,20].

**Figure 2:**
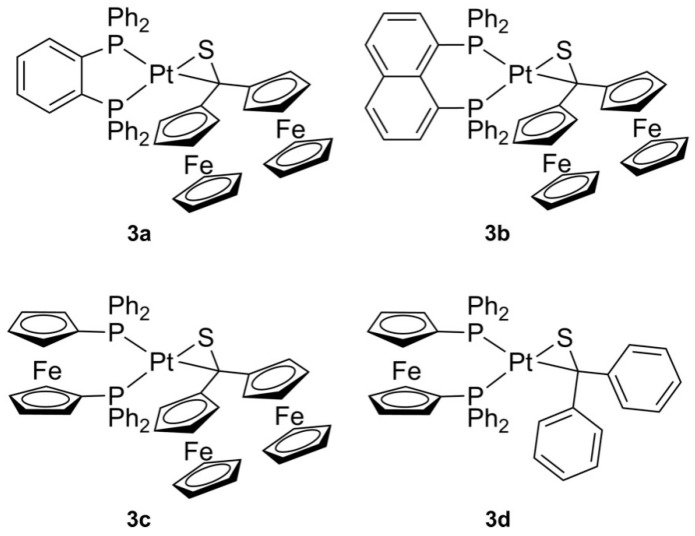
Platinathiiranes 3**a**–**d** investigated in this work.

*General procedure:* To a stirred solution of 0.063 mmole (bisphosphane)Pt(nbe) 2a–c [20] in 15 mL toluene one equivalent of thioketone **1a** (or **1b**) in toluene (10 mL) was added dropwise, whereupon the color of the solution changed. The mixture was stirred overnight at room temperature. Then, the solvent was removed under reduced pressure at room temperature and the solid residue was washed three to four times with diethylether. Next, the residue was dissolved in a small amount of toluene and subsequent diffusion of *n*-pentane into this concentrated solution gave crystals of complexes **3a**–**d** suitable for SC-X-ray diffraction. Structures of the synthesized complexes **3** are shown in Figure 2.

(dppbe)Pt(^2^-diferrocenylthioketone) **3a**. Orange colored crystals, yield: 61 mg (91%).^1^H (CD_2_Cl_2_, 400 MHz): 7.75 (m, 1H, dppbe), 7.65 (m, 5H, dppbe), 7.47 (m, 2H, dppbe), 7.39 (m, 6H, dppbe), 7.33 (m, 2H, dppbe), 7.23 (m, 8H, dppbe), 3.92 (m, 12H, 10H C_5_H_5_ and 2H C_5_H_4_), 3.85 (m, 2H, C_5_H_4_), 3.82 (m, 2H, C_5_H_4_), 3.75 (m, 2H, C_5_H_4_).^13^C{^1^H} (101 MHz, CD_2_Cl_2_): 134.1 (m) 133.9 (m), 133.6 (m), 133.1 (m), 132.7 (m), 131.8 (m), 131.2 (m), 130.6 (m), 130.2 (m), 128.9 (m), 128.6 (m), 102.9 (m, S=*C*Fc_2_), 70.3 (m), 69.7 (m), 69.4 (s, C_5_H_5_), 69.3 (m), 66.5 (s (C_5_H_4_), 66.3 (s, C_5_H_4_).^31^P{^1^H} NMR (CD_2_Cl_2_,162 MHz): 42.79 (d with ^195^Pt sat., ^1^*J*_PPt_ = 2835.0, ^2^*J*_PP_ = 28.1 Hz), 36.52 (d with ^195^Pt sat., ^1^*J*_PPt_ = 4167.2, ^2^*J*_PP_ = 28.0 Hz).^195^Pt{^1^H} (CD_2_Cl_2_, 129 MHz): −5059 (dd, ^1^*J*_PtP_ = 4168 Hz, ^1^*J*_PtP_ = 2835 MHz). ESI-MS(+mode) *m*/*z*: 1054.8 [M-2H]^+^ (including isotope pattern as calculated). EA: C_51_H_42_Fe_2_P_2_PtS**∙**0.5 toluene**∙**0.25 pentane (calc./found): C: 59.80/59.71; H: 4.41/4.40; S: 2.86/2.65.

(dppn)Pt(^2^-diferrocenylthioketone) **3b.** Orange colored crystals, yield: 47 mg (68%).^1^H (CD_2_Cl_2_, 400 MHz): 8.05 (m, 2H, *o*-H of dppn bridge), 7.42 (m, 2H, CH_ar_), 7.33 (m, 2H, CH_ar_), 7.25 (m, 8H, *o*-/*m*-C_6_H_5_), 7.14 (m, 8H, *o*-/*m*-C_6_H_5_), 6.91 (m, 4H, CH_ar_), 6.58 (CH_ar_), 4.20 (m, 10H, Cp C_5_H_5_), 4.12 (m, 2H, C*_p_* C_5_H_4_) 3.97 (m, 2H, C*_p_* C_5_H_4_), 3.90 (m, 2H, C*_p_* C_5_H_4_), 3.85 (m, 2H, C*_p_* C_5_H_4_). ^13^C{^1^H} (CD_2_Cl_2_, 101 MHz): 134.0 (m), 133.6 (m), 133.3 (m), 130.0 (m), 129.6 (m), 129.6 (m), 128.3 (m), 127.9 (m), 125.5 (m), 70.3 (m), 69.4 (m), 66.1 (m), 65.8 (m). ^31^P{^1^H} (CD_2_Cl_2_, 162 MHz): 4.63 (d with ^195^Pt sat. ^1^*J*_PPt_ = 2704 Hz, ^2^*J*_PP_ = 22.5 Hz), 4.26 (d with ^195^Pt sat. ^1^*J*_PPt_ = 3970 Hz, ^2^*J*_PP_ = 22.5 Hz). ESI-MS(+mode) *m*/*z*: 1104.6 [M-H]^+^ (including isotope pattern as calculated minus H). EA: C_55_H_44_Fe_2_P_2_PtS**∙**0.5 toluene**∙**0.25 pentane (calc./found): C: 59.74/59.15, H: 4.01/3.95, S: 2.90/2.62.

(dppf)Pt(^2^-diferrocenylthioketone) **3c**. Orange colored crystals, yield: 41 mg (56%). ^1^H (CD_2_Cl_2_, 400 MHz): 8.01 (m, 4H, C_6_H_5ar_), 7.5 (m, 6H, C_6_H_5_), 7.24 (m, 2H, C_6_H_5_), 7.03 (m, 8H, C_6_H_5_), 4.34 (m, 4H, C_5_H_4_), 4.19 (m, 12H, C_5_H_4_/C_5_H_5_), 4.09 (m, 2H, C_5_H_4_), 3.83 (m, 4H, C_5_H_4_), 3.74 (m, 2H, C_5_H_4_), 3.66 (m, 2H, C_5_H_4_). ^13^C{^1^H} (CD_2_Cl_2_, 101 MHz): 135.7 (s), 134.8–134.4 (m), 130.4 (m), 129.8 (m), 129.3 (m), 128.4 (m), 127.7 (m), 100.3 (m), 76.1 (m), 74.7 (m), 72.9 (m), 71.4 (m), 70.9 (m), 70.3 (m), 69.9 (m), 69.5 (m), 69.3 (m), 65.8 (m). ^31^P{^1^H} (CD_2_Cl_2_, 162 MHz): 17.66 (s with ^195^Pt sat. ^1^*J*_PPt_ = 4599 Hz), 14.14 (s with ^195^Pt sat. ^1^*J*_PPt_ = 3970 Hz). ^195^Pt{^1^H} (CD_2_Cl_2_, 129 MHz): −4900 (dd, ^1^*J*_PtP_ = 4597 Hz, ^1^*J*_PtP_ = 3117 MHz). ESI-MS(+mode) *m*/*z*: 1161.6 [M-2H]^+^ (including isotope pattern as calculated minus 2H). EA: C_55_H_46_Fe_3_P_2_PtS**∙**0.25 toluene (calc./found): C: 57.44/57.76; H: 4.08/4.11; S: 2.70/2.76.

(dppf)Pt(^2^-thiobenzophenone) **3d**. Colorless crystals, yield: 51 mg (85%). ^1^H (600 MHz, CD_2_Cl_2_): 7.91–7.83 (m, 4 H, CH_ar_), 7.45 (m, 6 H, CH_ar_), 7.29–7.20 (m, 6 H, CH_ar_), 7.05 (m, 8 H, CH_ar_), 6.92 (m, 4 H, CH_ar_), 6.89–6.85 (m, 2 H, CH_ar_), 4.16 (m, 12 H, C_5_H_4_ of cp). ^13^C{^1^H} (151 MHz, CD_2_Cl_2_): 134.7 (C_ar_), 134.7 (C_ar_), 134.4 (C_ar_), 134.4 (C_ar_), 130.5 (C_ar_), 130.1 (C_ar_), 130.1 (C_ar_), 128.5 (C_ar_), 128.4 (C_ar_), 128.0 (C_ar_), 127.9 (C_ar_), 127.1 (C_ar_), 124.2 (C_ar_), 75.9 (m, C_Cp_), 75.0 (s, C_Cp_), 73.0 (m, C_Cp_), 72.1 (s, C_Cp_). ^31^P{^1^H} (243 MHz, CD_2_Cl_2_): 18,82 (s with ^195^Pt-sat ^1^*J*_(PPt)_ = 4508 Hz), 17.64 (s with ^195^Pt-sat ^1^*J*_(PPt)_ = 3135 Hz) P–P coupling not resolute. ^195^Pt{^1^H} (129 MHz, CD_2_Cl_2_): -4988 (dd, ^1^*J*_(PPt)_ = 4511, 3132 Hz). MS (DEI): *m*/*z* (%) = 947 [M+] (8), 749 [M+-thioketone] (28), 198 [thioketone^+^] (98), 186 [ferrocene^+^] (99), 185 [diphenylphosphane^+^] (99), 165 [thioketone^+^-S] (100), 121 [thioketone^+^-phenyl] (100), 108 [phenylphosphane^+^] (99), 77 [phenyl^+^] (99). EA: C_47_H_38_FeP_2_PtS**∙**0,5CD_2_Cl_2_ (calc/found): C: 57.56/57.80; H, 4.07/4.30; S, 3.23/3,60.

### 2.2. Crystal Structure Determination

The intensity data for the compounds were collected on a NoniusKappaCCD (Bruker, Austin, TX, USA diffractometer using graphite-monochromated Mo-K_α_ radiation. Data were corrected for Lorentz and polarization effects; absorption was taken into account on a semi-empirical basis using multiple-scans [21,22,23]. The structures were solved by direct methods (SHELXS [23]) and refined by full-matrix least squares techniques against F_o_^2^ (SHELXL-97 [24] and SHELXL-2014 [25]). All hydrogen atoms of the compound **3b** were located by difference Fourier synthesis and refined isotopically. All other hydrogen atoms were included at calculated positions with fixed thermal parameters. The crystal of **3c** contains large voids, filled with disordered solvent molecules. The size of the voids is 192. Their contribution to the structure factors was secured by back-Fourier transformation using the SQUEEZE routine of the program PLATON [26] resulting in 40 electrons/unit cell. All non-disordered, non-hydrogen atoms were refined anisotropically [26]. Selected bond lengths (in Å) and angles (in °) measured for complexes **3a**–**c** using XRD methods are given in Table S1. Crystallographic data as well as structure solution and refinement details are summarized in Table S2 (Supporting Information). MERCURY [27] was used for structure representations.

*Supporting Information available*: Crystallographic data (excluding structure factors) has been deposited with the Cambridge Crystallographic Data Centre as supplementary publication CCDC-1922306 for **3a**, CCDC-1922307 for **3b**, and CCDC-1922308 for **3c**. Copies of the data can be obtained free of charge on application to CCDC, 12 Union Road, Cambridge CB2 1EZ, UK [E-mail: deposit@ccdc.cam.ac.uk].

### 2.3. Electrochemical Measurements

Cyclic voltammetry (CV) measurements were conducted using the three-electrode technique, with glassy carbon disk (diameter d = 1.6 mm) as the working electrode, Ag/Ag^+^ in acetonitrile (MeCN) as the reference electrode, and Pt wire as the counter electrode, with a Reference 600 Potentiostat (Gamry Instruments). Corrections for the iR drop were performed for all experiments. All experiments were performed in CH_2_Cl_2_ solutions (concentration of the complexes 1.0 mmol/L) containing 0.1 mol/L [nBu_4_N][BF_4_] at room temperature. Solutions were deaerated by N_2_ purge for 5–10 min, and a blanket of N_2_ was maintained over the solutions during the measurements. The glassy carbon disk was polished on a felt tissue with alumina before each measurement. All potential values reported in this paper are referenced to the potential of the ferrocenium/ferrocene (Fc^+^/Fc) couple. The dependence of the peak currents (I) from the square root of scan rate (v^1/2^) and logarithm of peak currents (log I) versus logarithm of scan rates (log v) for complexes **3a**–**d** (1.0 mmol/L) in CH_2_Cl_2_ - [nBu_4_N][BF_4_] (0.1 m/L) are given in Figure S1.

### 2.4. Theoretical Calculations

The structure of complex **3c** was optimized at the BP-D/def2-TZVP level of theory [28,29,30,31,32,33,34,35] (Tables S3 and S4). The XRD geometry of **3c** was used as a starting point for the optimization (Figure S2). Vibrational frequency calculations confirmed that the optimized geometry corresponded to a true minimum on the potential energy surface of **3c**. The same calculations were also carried out for the separate **1a** and (dppf)Pt fragments constituting **3c**. All these calculations were done with the TURBOMOLE 7.2 program [36]. For the optimized complex, a single-point energy calculation with a slightly larger basis set (def2-TZVPP) [34] was performed using Gaussian 09 D.01 [37]. This program allowed to generate the molecular wave function files required to analyze the bonding situation in terms of natural bond orbitals (NBO) [38] and the quantum theory of atoms in molecules (QTAIM) [39]. The NBO analysis was done with the NBO 3.1 subroutine [40] incorporated into Gaussian 09 D.01. This subroutine was also used by Multiwfn 3.5 [41] to express the composition of frontier molecular orbitals in terms of natural atomic orbitals [42]. In the case of QTAIM, its implementation available in AIMAll 14.11.23 [43] was utilized. To verify the results obtained from BP-D/def2-TZVPP, an additional single-point energy calculation was carried out using the popular B3LYP hybrid density functional [44,45,46] combined with the def2-TZVPP basis set (these results are presented in Tables S3 and S4 and in Figures S3 and S4). Results from both density functionals turned out to be in qualitative agreement, and therefore, only the BP-D results will be quoted in Section 3.3. Cartesian coordinates (in Å) for **3c**, **1a**, and (dppf)Pt-fragment are collected in Tables S5–S7, respectively (the SI part, see S4).

## 3. Results and Discussion

### 3.1. Synthesis and Characterization of Platinathiiranes ***3a**–**d***

Acetylation of ferrocene with ferrocenyl carboxylic acid using the so-called “mixed anhydride” prepared in situ by treatment of the latter with trifluoroacetic anhydride (TFAA) opens a straightforward access to the required diferrocenyl ketone. Subsequent thionation of this ketone with Lawesson´s reagent in toluene solutions leads to the desired thioketone **1a** in high yield [9].

The test experiment for the preparation of platinathiiranes was performed starting with **1a** and **2a** in toluene solution at room temperature using equimolar amounts of both compounds. After 24 h the solvent was removed and a crystalline product was obtained. The ^1^H NMR spectroscopic analysis showed a set of signal characteristic for aromatic CH units as well as for two chemically equivalent ferrocenyl groups.

**Scheme 3:**
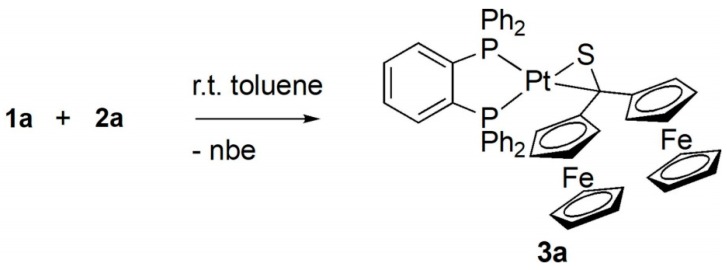
Synthesis of the platinathiirane complex **3a** derived from **1a** and **2a**.

In the ^13^C{^1^H} NMR spectrum the signal located at 102.9 ppm confirmed the presence of the C–S moiety. In comparison with the resonance signal of **1a** (237.1 ppm) the ^13^C signal of the thiocarbonyl group, was drastically shifted to lower frequencies. Finally, the registered ^31^P{^1^H} NMR spectrum revealed an AB spin system at 42.79 and 28.10 ppm with ^1^J(PtP) = 2835 and 4167 Hz, respectively. In addition, the ESI-MS showed the *m*/*z* = 1054.8 which corresponds to the calculated [M–2H]^+^ ion for the expected 1:1 complex **3a** (Scheme 3).

**Figure 3:**
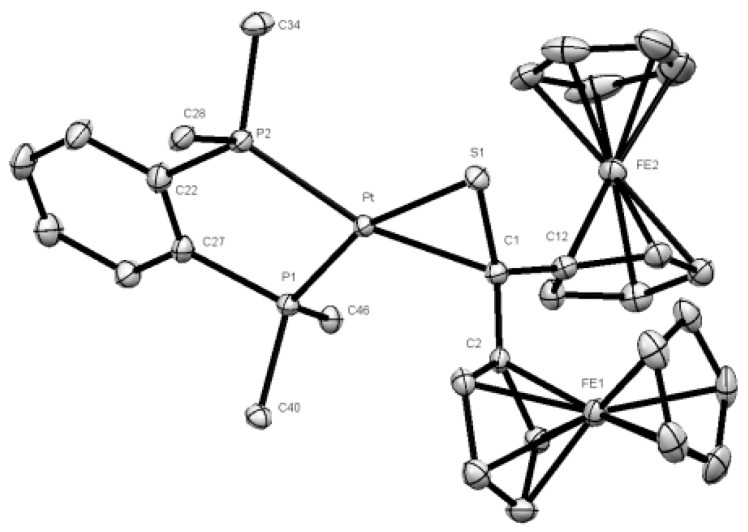
Molecular structure (50% probability) of complex **3a**. Hydrogen atoms, solvent molecule (toluene), as well as the phenyl rings of the dppbe ligand are omitted for clarity.

The obtained product **3a** was crystallized by diffusion of pentane into the concentrated toluene solution and single crystals suitable for XRD analysis were obtained. The analysis confirmed unambiguously the proposed structure of platinathiirane **3a** (Figure 3).

**Figure 4:**
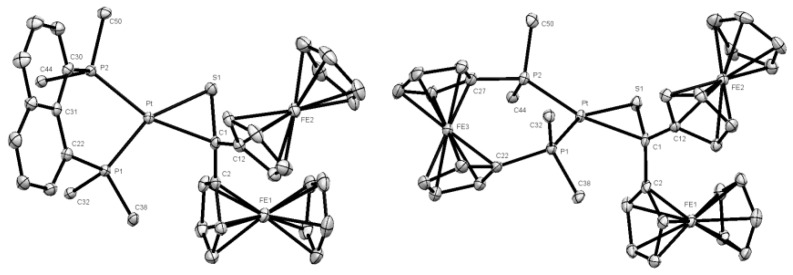
Molecular structures (50% probability) of complexes **3b** (**left**) and **3c** (**right**). Hydrogen atoms, solvent molecule (CH_2_Cl_2_) (in **3b**) as well as the phenyl rings of the dppn (**3b**) and dppf (**3c**) ligands are omitted for clarity.

The bond lengths Pt-S(1) [2.3014(8) Å], S(1)-C(1) [1.791 (3) Å], and Pt-C(1) [2.155(3) Å] (see Table S1) are comparable with values reported for analogous platinathiiranes containing the same bisphosphane ligand [18,47]. The same method was used for the preparation of platinathiiranes **3b** and **3c** starting with **1a** and Pt(0) complexes **2b** and **2c**, respectively. The structures of complexes **3b** and **3c** were also established by means of spectroscopic methods as well as by XRD analysis (Figure 4).

In extension of the study, reaction of **1b** with complex **2c** was also carried out and the expected platinathiirane **3d** was isolated in 85% yield and collected spectroscopic data confirmed unambiguously its structure (see Experimental). 

### 3.2. Electrochemical Studies

**Figure 5:**
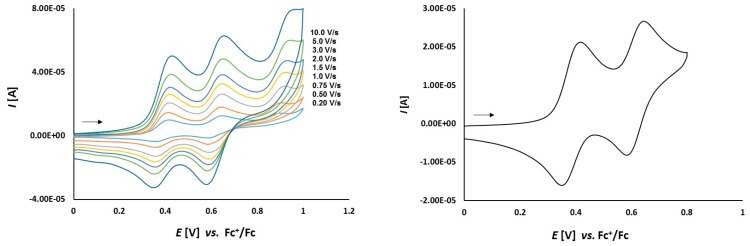
Cyclic voltammetry of complex **1a** (1.0 mmol/L) in CH_2_Cl_2_-[*n*Bu_4_N][BF_4_] (0.1 m/L) solutions at various scan rates (**left**) and at v = 0.75 V/s (**right**). The potentials *E* are given in *V* and referenced to the Fc^+^/Fc couple. The arrows indicate the scan direction.

**Figure 6:**
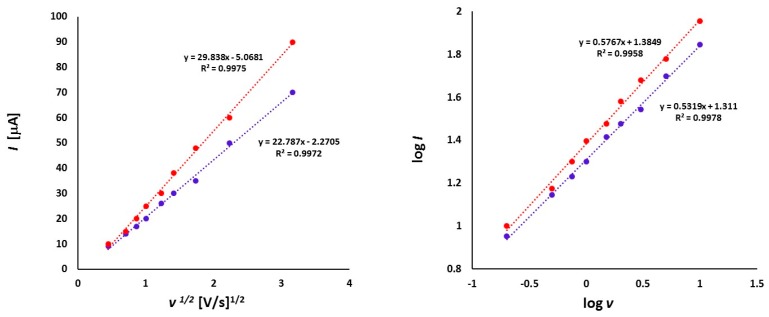
The dependence of the peak currents (*I*) from the square root of scan rate (*v^1/2^*) (**left**) and logarithm of peak currents (log *I*) versus logarithm of scan rates (log *v*) (**right**) for **1a** (1.0 mmol/L) in CH_2_Cl_2_ - [*n*Bu_4_N][BF_4_] solution (0.1 m/L).

**Figure 7:**
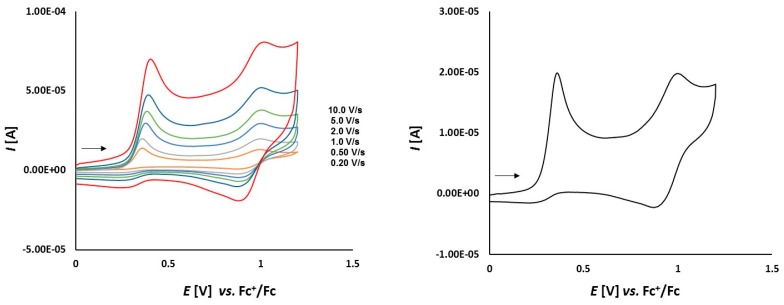
Cyclic voltammetry of complex **3d** (1.0 mmol/L) in CH_2_Cl_2_-[*n*Bu_4_N][BF_4_] (0.1 m/L) solutions at various scan rates (**left**) and at *v* = 0.50 V/s (**right**). The potentials *E* are given in *V* and referenced to the Fc^+^/Fc couple. The arrows indicate the scan direction.

**Figure 8:**
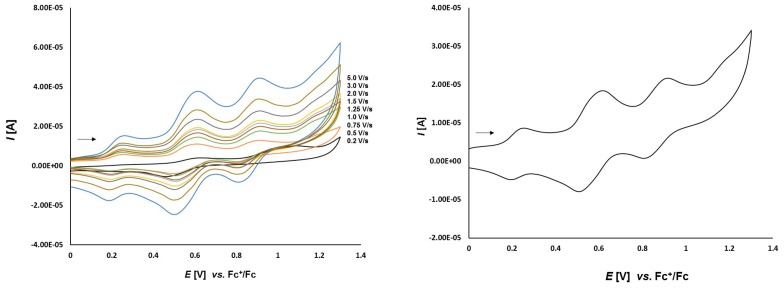
Cyclic voltammetry of complex **3a** (1.0 mmol/L) in CH_2_Cl_2_-[*n*Bu_4_N][BF_4_] (0.1 m/L) solutions at various scan rates (**left**) and at *v* = 1.50 V/s (**right**). The potentials *E* are given in *V* and referenced to the Fc^+^/Fc couple. The arrows indicate the scan direction.

**Figure 9:**
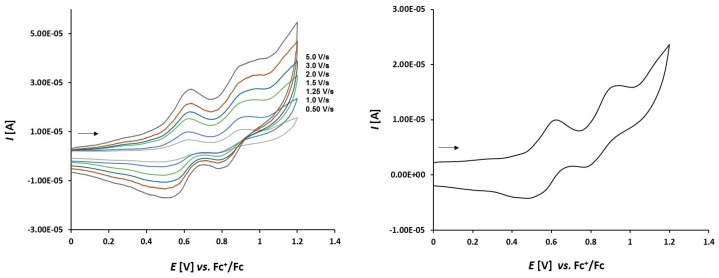
Cyclic voltammetry of complex **3b** (1.0 mmol/L) in CH_2_Cl_2_-[*n*Bu_4_N][BF_4_] (0.1 m/L) solutions at various scan rates (**left**) and at *v* = 1.0 V/s (**right**). The potentials *E* are given in *V* and referenced to the Fc^+^/Fc couple. The arrows indicate the scan direction.

**Figure 10:**
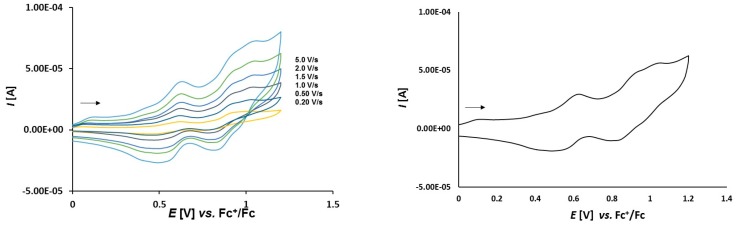
Cyclic voltammetry of complex **3c** (1.0 mmol/L) in CH_2_Cl_2_-[*n*Bu_4_N][BF_4_] (0.1 m/L) solutions at various scan rates (**left**) and at *v* = 2.0 V/s (**right**). The potentials *E* are given in *V* and referenced to the Fc^+^/Fc couple. The arrows indicate the scan direction.

In the second part of the study, the obtained platinathiiranes **3a**–**d** were investigated by CV in CH_2_Cl_2_ solutions. For comparison, the cyclic voltammogram of thioketone **1a** was also recorded and analyzed (Figure 5 and Figure 6). Cyclic voltammograms of complexes **3a**–**d** are shown in Figure 7, Figure 8, Figure 9 and Figure 10. 

#### 3.2.1. Thioketone **1a**

The electrochemical behavior of biferrocene can be affected by at least two types of interaction between the two ferrocene moieties [48]. The first is the interaction through the bridge atoms, and the second is the interaction between two iron–iron centers.

Biferrocene undergoes two reversible, one-electron oxidation processes with the separation potential value of 340 mV [49,50]. These two distinct one-electron oxidations can be explained by the fact that cyclopentadienyl rings around the Fe(1) and Fe(2) are differently organized. The rings around the Fe(1) atom are eclipsed, whereas these around Fe(2) are staggered. Because of this orientation the Fe–cyclopentadienyl rings mean bond lengths are different in case of Fe(1) and Fe(2) which results in electronic delocalization and the biferrocenium ion can be classified as a mixed-valent (Fe^II^-Fe^III^) compound displaying localized charge. Both Fe atoms are in different oxidation states, Fe(II) and Fe(III), respectively. Thus the first oxidation process removes an electron from the Fe(II).

In diferrocenylmethane the separation potential value is of 120 mV [51]. This may point to less communication between the two ferrocene moieties. But if we look at the two-bridged [1,1]-ferrocenephane, the separation between oxidation potentials is about twice as large, 190 mV [48]. The authors assume that difference in that case is due to a direct Fe–Fe electrostatic field interaction which may increase the communication in case of [1,1]-ferrocenophane where the rotation around –CH_2_– function is suppressed. This assumption can be also supported by the structure in which both of the iron atoms are placed off-center in the ferrocene moieties. In such configuration the intramolecular repulsion of the nonbonding electrons of iron atoms can be reduced [52].

1,2-Diferrocenylethane displays in dichloromethane solution a single two-electron oxidation process which is reversible [51]. This can be considered as an evidence for the lack of any electronic communication between the two ferrocenyl moieties.

However, for 1,2-diferrocenylethene and diferrocenylethyne the second oxidation peak appears again and the separation potential values are of 160 and 140 mV, respectively. Thus the conjugated unsaturated C=C and C≡C bonds allow again for the electronic communication between these two moieties [51,53].

As it is shown in Figure 5, thioketone **1a** in dichloromethane solution, similarly to biferrocene [48], undergoes two distinct one-electron oxidations. Both signals correspond to the oxidation of the two iron atoms in the ferrocene moieties. First oxidation occurs at *E*_ox1_ = 0.164 V and the corresponding reduction is observed at *E*_red1_ = 0.099 V. The second oxidation occurs at *E*_ox2_ = 0.392 V with the corresponding reduction peak at *E*_red2_ = 0.332 V. Both red-ox systems are reversible because of the peak-to-peak separation which is Δ*E*_1_ = 65 mV and Δ*E*_2_ = 60 mV, respectively, as well as the *I*_ox_/*I*_red_ ratios close to 1.

The fact that the peak-to-peak separation Δ*E* values slightly departed from the theoretical value of 59 mV, seem to contrast with diagnostic criteria for an electrochemically reversible one-electron electrode process. Not completely compensated resistance given by the applied solvent and supporting solution can explain this fact.

The dependence of scan rate measurements showed that the peak currents for each peak varied linearly with the square root of scan rate (*v*^1/2^) (Figure 6). It can point to the diffusion controlled process. Also, the dependence of logarithm of peak currents versus logarithm of scan rates displayed straight lines with slope values 0.5319 and 0.5767, respectively, that were close to the theoretical value of 0.5 which is expected for a diffusion controlled electrode process.

**Scheme 4:**
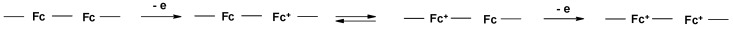
The postulated electrode reaction processes of **1a**.

The fact that **1a** undergoes two separate one-electron oxidation (Scheme 4) suggests a similar course of electrochemical processes occurring in biferrocene [49,50] and diferrocenyl ketone [54,55] as well. In analogy to biferrocene molecule, the Fe–cyclopentadienyl rings mean bond lengths are also different in case of Fe(1) and Fe(2). This might result in the electronic delocalization and the cation derived from **1a** can be regarded also as a mixed-valent (Fe^II^-Fe^III^) compound. In case of diferrocenylketone and **1a** the electronic delocalization can be enhanced by the presence of C=O and C=S functionalities, respectively.

The degree of charge delocalization in mixed-valent **1a** can be determined by calculation of the comproportionation constant *K*_comp_ as presented in Equations (1)–(4):

*K*_comp_ = 10^(*F*×^^Δ*E*/2.303×*RT*)^ (1)

at 25 °C *K*_comp_ = 10^(38.92×^^Δ*E*/2.303)^ = 10^(16.90×^^Δ*E*)^ (2)

for Δ*E*^0’^ = 0.392_(*E*ox2)_ V − 0.164_(*E*ox1)_ V = 0.228 V (3)

*K*_comp_ = 10^(16.90×^^Δ*E*)^ = 10^(16.90×0.228)^ = 10^3.8532^ (4)

According to Robin-Day classification [54], if *K*_comp_ value is within the range 10^2^–10^6^ the compound belongs to class II in which the charge is slightly delocalized between the two red-ox centers.

An oxidation of the thiocarbonyl functionality was not observed within applied potential range. Thus, in contrast to ketones, the ferrocenyl moiety seems to stabilize the thiocarbonyl functionality in the molecule of ferrocenyl functionalized thioketones [53].

#### 3.2.2. Platinathiiranes **3a**–**d**

Complex **3d** (Figure 7), bearing the ferrocene moiety in the bridging bisphosphane ligand shows an additional oxidation process at 0.757 V and reduction at 0.641 V (Δ*E*_p_ = 116 mV). This irreversible process can be assigned to the dppf ligand. To prove this we also measure the electrochemical behavior of **2c** which shows a reversible oxidation event at 0.651 V. It can be seen as the reversible oxidation of the ferrocene function in the dppf ligand chelating the platinum center. Its difference to the potential of the reversible oxidation of **3d** is caused by the different π-backbonding of the norbornene to the (bisphosphane)Pt complex fragment.

The electrochemical properties of complexes **3a**–**c** show a much more reversible character. The voltammograms of complex **3a** depicted in Figure 8 show three full reversible oxidation events at 0.003 V, 0.363 V, and 0.668 V. The reversible oxidation of two ferrocene moieties at the thioketone site can be observed at 0.363 V and 0.668 V, respectively. The ferrocene moieties at the thioketone site seem to stabilize the oxidized intermediates of the sulfur and a chemical reaction leading to thiosulfine as oxidation products are hindered.

On the voltammograms of complex **3b** presented in Figure 9 three reversible redox systems can be observed. The first well-defined oxidation at 0.390 V is followed by two poorly separated signals at 0.650 V and 0.750 V, respectively. In comparison to other compounds these signals correspond to the oxidation of the two ferrocene moieties of the thioketone ligand. Remarkably, oxidation of the sulfur atom is not observed in this complex.

The cyclic voltammograms of **3c** presented in Figure 10 show three oxidation events at 0.387 V, 0.693 V, and 0.797 V. The first two events represent the oxidation of the two ferrocenes at the thioketone site and the last event at 0.797 V is attributed to the oxidation of the dppf ligand.

In complexes **3a**–**c** the dependence of scan rate measurements (see Figure S4) show that the peak currents for each peak vary linearly with the square root of scan rate (*v*^1/2^) which points out to the diffusion controlled processes. The plots of logarithm of peak currents versus logarithm of scan rates display straight lines with slope values within the range 0.8–0.6 which points out rather to mixed diffusion and adsorption controlled electrode process.

### 3.3. Computational Studies

The calculated structure of complex **3c** is close to its geometry determined by the XRD measurement (see Figure S2). The experimental bond lengths and bond angles are reproduced very well. The root-mean-square deviation (RMSD) of the calculated bond lengths from the corresponding experimental data amounts to 0.065 Å. Even smaller deviation can be reached for the bond lengths involving only non-hydrogen atoms (RMSD = 0.010 Å). Similarly, the calculated bond angles differ from the corresponding experimental values by no more than 0.92° on average (RMSD = 1.01° for the bond angles involving only non-hydrogen atoms). The torsion angles of the optimized complex show however significant deviations from their XRD values (RMSD = 15.0° and 18.1° for the torsion angles involving all and only non-hydrogen atoms, respectively). The greatest differences occur for the torsion angles defining the spatial arrangement of all phenyl rings and two ferrocenyl groups at the C(1) atom. These groups are located further away from the rigid core of **3c** and their torsion angles are softer. In consequence, they are most affected by crystal packing effects, as it is manifested by the XRD structure. The optimized geometry of the complex **3c** displays an almost planar fragment containing the Pt center and its four adjacent atoms [that is, P(1), P(2), S(1), and C(1)].The calculated bond lengths and bond angles in the immediate neighborhood of the Pt center are listed in Table 1. The calculated bond lengths and angles involving the Pt center differ by no more than 0.032 Å and 3.4° from the corresponding experimental results. The Pt‒P(1) and Pt‒P(2) bonds are elongated slightly upon complexation of (dppf)Pt with **1a**. Similarly, the C=S bond of **1a** becomes longer if coordinated by the Pt center of (dppf)Pt.

Table 2 presents the Wiberg bond index (WBI) [56] and the delocalization index (DI) [57], which are obtained from the NBO and QTAIM calculations, respectively. The elongations of the Pt‒P(1), Pt‒P(2), and C(1)‒S(1) bonds in **3c** are associated with decreases in their bond order indices. Both WBI and DI suggest that the Pt‒S(1) bond is essentially similar to Pt‒P(1) and Pt‒P(2). Compared to these bonds, the Pt‒C(1) bond is characterized by lower values of WBI and DI. This implies smaller strength and covalent character for the Pt‒C(1) bond. The formation of the complex results in particularly significant decreases in the WBI and DI values of the C(1)‒S(1) bond. These indices reveal that the C(1)‒S(1) bond order loses its formally double character and this bond actually becomes close to a single bond (a typical single C‒S bond, such as that in *tert*-butylthiol, shows the WBI and DI values of 0.974 and 1.023, respectively).

The bonds around the Pt center are further characterized using QTAIM parameters determined at bond critical point (BCP). Bond paths and associated BCPs were found for all four bonds involving the Pt center. The values of several important QTAIM parameters at the BCPs are listed in Table 3.

In general, the QTAIM characteristics of the Pt‒P(1), Pt‒P(2), Pt‒S(1), and Pt‒C(1) bonds are rather typical of metal-ligand bonds [58,59,60]. According to QTAIM, metal-ligand bonds are classified as interactions falling in the transit zone between shared (that is, classical covalent) and purely closed shell (that is, ionic) interactions [61]. On the one hand, the BCPs of Pt‒P(1), Pt‒P(2), Pt‒S(1), and Pt‒C(1) show relatively small values of the electron density (*ρ*) and the positive sign of the Laplacian of *ρ* (∇^2^*ρ*) [61]. Such features reveal the closed shell character of interactions. On the other hand, the total energy density (*H*) at these BCPs adopts negative values, which in turn implies a covalent contribution to bonding in Pt‒P(1), Pt‒P(2), Pt‒S(1), and Pt‒C(1) [62]. The presence of covalent contribution is confirmed by two other BCP parameters: the ratio of the potential energy density (*V*) to the kinetic energy density (*G*) and the ratio of two Hessian eigen values (*λ*_1_,*λ*_3_). For the four bonds, their values of ‒*V*/*G* are between 1 and 2 and ‒*λ*_1_/*λ*_3_ > 0.25. Such ranges of ‒*V*/*G* and ‒*λ*_1_/*λ*_3_ values indicate some degree of covalency between bonded atoms [63,64]. The BCP of the C(1)‒S(1) bond in both the complex and **1a** possesses the features of typical shared interactions (a high value of *ρ*, ∇^2^*ρ* < 0, *H* < 0). Furthermore, its QTAIM parameters demonstrate a decrease in electron sharing between C and S upon complexation with (dppf)Pt.

**Figure 11:**
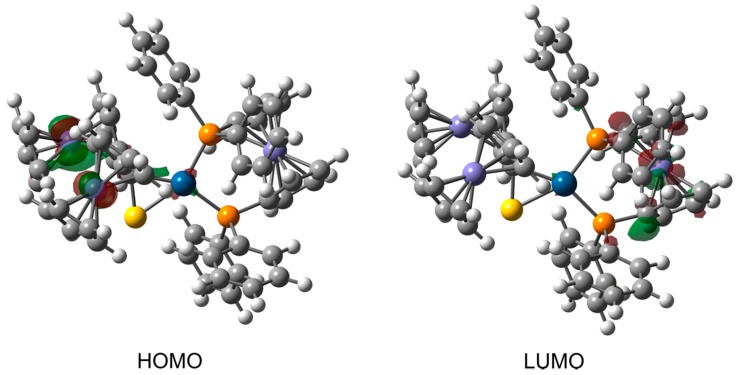
Contours of the highest occupied molecular orbital and lowest unoccupied molecular orbital for complex **3c**. Their parts possessing either positive or negative sign are colored red and green, respectively. The contours are plotted with an isovalue of 0.05 au.

Contours of frontier molecular orbitals for **3c** are depicted in Figure 11. The main part of the highest occupied molecular orbital (HOMO) is localized around the Fe atoms of thioketone fragment. The contribution of Pt orbitals to the HOMO is also evident. In general, both the HOMO and its nearest orbitals with lower energies (e.g., HOMO-1, HOMO-2, HOMO-3, see Figure S3) are largely composed of the Fe orbitals of thioketone fragment. 

In contrast to the HOMO, the lowest unoccupied molecular orbital (LUMO) is mainly concentrated on the ferrocenyl and phenyl groups of (dppf)Pt fragment. Similarly, the molecular orbitals above the LUMO also show significant contributions from these groups (see Figure S4). The complex exhibits a HOMO-LUMO gap of 1.82 eV.

In addition to the visual inspection of HOMO and LUMO contours, the frontier molecular orbitals of **3c** are also analyzed quantitatively by expressing their percentage composition in terms of natural atomic orbitals (NAOs). The HOMO mainly consists of the valence 3d NAOs belonging to two Fe atoms of thioketone fragment. The percentage contributions from the NAOs of these Fe atoms sum up to ca. 73% in the HOMO. The leading contribution of ca. 29% originates from the 3d_yz_ orbital of one of the Fe atoms. Next relevant contributions, but much smaller in magnitude, are ascribed to the NAOs of the Pt and S(1) atoms. These atoms make similar contributions, each adds up to ca. 3%. The LUMO shows a significant contribution of the 3d_x2−y2_ NAO belonging to the Fe atom of (dppf)Pt fragment. This NAO constitutes ca. 21% of the LUMO and it is by far the largest individual NAO contribution. In total, the Fe atom of (dppf)Pt makes a contribution of ca. 32%. The rest of the LUMO also comes from the (dppf)Pt fragment, in particular from the NAOs of C atoms belonging to its ferrocenyl and phenyl groups.

The NBO analysis carried out for **3c** reveals that there is only one bonding NBO in the thiocarbonyl group of thioketone fragment. Within the framework of the NBO method, bonding and back-bonding contributions to metal-ligand bonds can be described by electron delocalizations between donor and acceptor NBOs (such delocalizations can be further characterized by the respective stabilization energies derived from the second-order perturbation theory of NBO donor-acceptor interactions [38]). Donor-acceptor delocalizations from the bonding NBO of C(1)‒S(1) to the lone pair and Rydberg NBOs of the Pt center have indeed been detected in **3c** but their stabilization energies are not dominating (ca. 55 kcal/mol in total). Very small stabilization energies (ca. 9 kcal/mol in total) are attributed to the delocalizations from the valence NBOs of Pt to the antibonding NBO of C(1)‒S(1). In this case, the valence NBOs of Pt include its 5d_z2_ and 5d_x2−y2_ orbitals.

It is instructive to relate the above computations to the results of CV measurements for **3c**. As it was mentioned above, the HOMO of the complex is mainly localized on two Fe atoms of thioketone fragment, but their contributions to the HOMO are by no means equal. This proves the CV assignment of two lowest oxidation events to two ferrocene moieties at the thioketone site of **3c**. For various diferrocenyl derivatives it was reported that the separation between their oxidation potentials is often associated with the distance between their Fe atoms [55]. The separation tends to increase as the Fe–Fe distance decreases. For thioketone **1a**, its Fe–Fe distance and oxidation potential separation amount to 5.207 Å and 0.228 V, respectively. For complex **3c** in its optimized geometry, the distance between two Fe atoms of the thioketone fragment is 5.125 Å and the value of 0.306 V was measured as the separation between two lowest oxidation potentials. Accordingly, the Fe–Fe distance is smaller for **3c**, and therefore, the separation between its two lowest oxidation potentials grows, compared to **1a**.

## 4. Conclusions

The present study showed that diferrocenyl thioketone (**1a**) reacts easily with (bisphosphane)Pt(0) complexes **2a**–**c**, yielding platinathiiranes **3a**–**c**. In contrast to complexes **3a**,**b**, complex **3c** possesses an additional ferrocenyl group which acts as the backbone of the bisphospohane ligand. In addition, platinathiirane **3d** was prepared from thiobenzophenone (**1b**) and **2c**. Three of the four complexes **3a**–**d** have been characterized using XRD methods revealing that the bond lengths in the platinathiirane rings correspond to the data reported for similar platinaheterocycles derived from several aryl and cycloaliphatic thioketones [16,17,18,47]. Compounds **3a**–**d** were characterized electrochemically using CV. For the sake of comparison, the starting thioketone **1a**, was also studied electrochemically. Complexes **3a**–**c** derived from **1a** behaved similarly and exhibit reversible oxidation processes. Together with **3d**, these complexes undergo a mixed diffusion/adsorption controlled electrode process, whereas **1a** is oxidized by a diffusion controlled mechanism. The quantum-chemical computations performed for **3c** revealed that the C=S bond of **1a** lost its formally double character upon complexation with (dppf)Pt and its bond order became quite close to unity. 

The chemistry of metallathiiranes and related metallthiirenes attracts considerable attention of many groups and the nature of the metal-C=S bond in these compounds is a problem of current interest [65]. In addition, because of the well-documented importance of ferrocenyl functionalized compounds, the new complexes **3a**–**d** offer a novel type of ferrocene- and platinum-containing sulfur systems for potential applications in material chemistry [66]. Notably, some of the Pt sulfur/nitrogen complexes were reported to induce stereoselective polymerization in methylthiirane [67] but in general, the problem of potential application of sulfur–platinum complexes in polymer chemistry is lesser known and requires further studies.

In the final conclusion it should be also stressed that the present study evidences one more time the importance of thioketones for organometallic and coordination chemistry. In recent two decades they were demonstrated to act as perfect complexation reagents for diverse metals at variable oxidation states, such as platinum, ruthenium, tungsten, etc., [68]. For that reason, ferrocenyl functionalized thioketones reported in the study should be also regarded as potentially useful trapping reagents not only for Pt(0) complexes but also for other metals. Potentially, they can also be applied in reactions with iron carbonyls aimed at the preparation of new redox-active iron–sulfur clusters recognized as hydrogenase mimics [69,70,71,72].

## Figures and Tables

**Table 1:** Calculated coordination geometry around the Pt center in complex **3c**. For some of the geometrical parameters, their values calculated for the isolated **1a** and (dppf)Pt molecules are presented in parentheses.

Atoms	Bond Length (Å)	Atoms	Bond Angle (°)
Pt‒P(1) Pt‒P(2) Pt‒S(1) Pt‒C(1) C(1)‒S(1)	2.246 (2.213) 2.282 (2.213) 2.326 2.155 1.788 (1.665)	P(1)‒Pt‒P(2) P(1)‒Pt‒C(1) P(2)‒Pt‒S(1) C(1)‒Pt‒S(1)	99.8 (129.1) 105.7 107.7 46.8

**Table 2:** Calculated natural bond orbitals (NBO) and quantum theory of atoms in molecules (QTAIM) indicators of bond order for the bonds around the Pt center in complex **3c**. For some of the bonds, their order indices calculated for the isolated **1a** and (dppf)Pt are presented in parentheses.

Atoms	WBI	DI
Pt‒P(1) Pt‒P(2) Pt‒S(1) Pt‒C(1) C(1)‒S(1)	0.654 (0.835) 0.636 (0.835) 0.689 0.417 1.148 (1.656)	1.057 (1.1865) 0.999 (1.1869) 0.926 0.701 1.157 (1.696)

**Table 3:** Calculated QTAIM parameters of bond critical points for the bonds around the Pt center in complex **3c**. For some of the bonds, their QTAIM parameters calculated for the isolated **1a** and (dppf)Pt are presented in parentheses.

Atoms	*ρ* (au)	∇^2^*ρ* (au)	*H* (au)	‒*V*/*G*	‒*λ*_1_/*λ*_3_
Pt‒P(1)	0.1232 (0.1278)	0.0755 (0.0584)	−0.0642 (−0.0712)	1.7733 (1.8305)	0.3711 (0.4011)
Pt‒P(2)	0.1151 (0.1279)	0.0911 (0.0584)	−0.0557 (−0.0713)	1.7104 (1.8306)	0.3413 (0.4011)
Pt‒S(1) Pt‒C(1) C(1)‒S(1)	0.1010 0.1100 0.1839	0.1457 0.1239 −0.2656	−0.0401 −0.0454 −0.1349	1.5242 1.5951 2.9701	0.2912 0.3625 1.1881
	(0.2269)	(−0.3901)	(−0.2612)	(2.5958)	(2.1543)

## Supplementary Materials

The following are available online at https://www.mdpi.com/1996-1944/12/17/2832/s1, Section S1: Selected bond lengths and angles measured for complexes **3a**–**c** using XRD methods (Table S1) and crystal data and refinement details for the X-ray structure determinations (Table S2). Section S2: Further electrochemical details: The dependence of the peak currents (*I*) from the square root of scan rate (*v^1/2^*) and logarithm of peak currents (log *I*) versus logarithm of scan rates (log *v*) for complexes **3a**–**d** (1.0 mmol/L) in CH_2_Cl_2_-[*n*Bu_4_N][BF_4_] (0.1 m/L) (Figure S1). Section S2: Further computational details. Table S3: Calculated NBO and QTAIM indicators of bond order for the bonds around the Pt center in complex **3c**. Table S4: Calculated QTAIM parameters of bond critical points for the bonds around the Pt center in complex **3c**. Figure S2: Superposition of the calculated and experimental structures of complex **3c**. Figure S3: Contours of several molecular orbitals below and above the frontier molecular orbitals for complex **3c**. Figure S4: Contours of the highest occupied molecular orbital and lowest unoccupied molecular orbital for complex **3c**. Section S4: Cartesian coordinates (in Å) for **3c**, **1a**, and (dppf)Pt-fragment: Tables S5–S7.

## References

[1] Voss J. (1985). Thioaldehyde bzw. Thioketone. Houben-WeylMethoden der Organischen Chemie: Organische Schwefel-Verbindungen.

[2] Paquer D. (1972). Aliphatic Thioketones. Int. J. Sulfur Chem. B.

[3] Ohno A., Oae S. (1977). Thiones. Organic Chemistry of Sulfur.

[4] Mlostoń G., Grzelak P., Hamera-Fałdyga R., Jasiński M., Urbaniak K., Pipiak P., Albrecht Ł., Hejmanowska J., Heimgartner H. (2017). Aryl, hetaryl, and ferrocenylthioketones as versatile building blocks for exploration in the organic chemistry of sulfur. Phosphorus Sulfur Silicon Relat. Elem..

[5] Huisgen R., Langhals E. (2006). 1,3-Dipolar cycloadditions of diphenyldiazomethane to thioketones: Rate measurements disclose thiones to be superdipolarophiles. Heteroat. Chem..

[6] Rohr U., Schatz J., Sauer J. (1998). Thio-and selenocarbonyl compounds as “Superdienophiles” in cycloadditions. Eur. J. Org. Chem..

[7] Sugiyama N., Yoshioka Y., Aoyama H., Nishio T. (1971). Photochemical reaction of thioacetophenone trimer (2,4,6-trimethyl-2,4,6-triphenyl-1,3,5-trithiolan). J. Chem. Soc. D.

[8] Mlostoń G., Hamera-Fałdyga R., Linden A., Heimgartner H. (2016). Synthesis of ferrocenyl-substituted 1,3-dithiolanes via [3 + 2]-cycloadditions of ferrocenyl hetaryl thioketones with thiocarbonyl S-methanides. Beilstein J. Org. Chem..

[9] Mlostoń G., Hamera R., Heimgartner H. (2015). Synthesis of ferrocenyl thioketones and their reactions with diphenyldiazomethane. Phosphorus Sulfur Silicon Relat. Elem..

[10] Sato M., Asai M. (1992). Synthesis and some properties of diferrocenyl thioketones and dynamic behavior of some [1.1] ferrocenophane derivatives. J. Organomet. Chem..

[11] Rausch M.D., Fischer E.O., Grubert H. (1960). The aromatic reactivity of ferrocene, ruthenocene and osmocene. J. Am. Chem. Soc..

[12] Astruc D. (2017). Why is ferrocene so exceptional?. Eur. J. Inorg. Chem..

[13] Kowalski K., Karpowicz R., Mlostoń G., Miesel D., Hildebrandt A., Lang H., Czerwoniec R., Therrien B. (2015). Synthesis and (spectro)electrochemistry of mixed-valent diferrocenyl-dihydrothiopyran derivatives. Dalton Trans..

[14] Kursanov D.N., Setkina V.N., Dolgova S.P., Nefedova M.N. (1980). Preparation of heterometallic binuclear complexes of transition metals from metallocenyl thioketones. Izv. Akad. Nauk. SSSR Ser. Khim..

[15] Barnes J.C., Bell W., Glidewell C.h., Howie R.A. (1990). Metal complexation of thioacylferocenes: Crystal structure of pentacarbonyl (thiobenzoylferrocene-S) chromium and benzoylferocene. J. Orgnomet. Chem..

[16] Weigand W., Wünsch R., Robl C., Mlostoń G., Nöth H., Schmidt M. (2000). Metallkomplexe mit funktionalisierten Schwefelliganden, XV [1]. Reaktionen von Platin(0)-Komplexen mit 1,2,4-Trithiolanen, 1,2,4,5-Tetrathianen, 1,2,3,5,6-Pentathiepanen sowie Thioketonen. Kristallstrukturanalyse von (Ph_3_P)_2_Pt(*η*^2^-Ph_2_C = S). Z. Naturforsch. B.

[17] Weisheit T., Kritz A., Görls H., Mlostoń G., Imhof W., Weigand W. (2012). Reaction of ‘non-symmetrical’ 1,2,4-trithiolanes with phosphanePt^0^(*η*^2^-nbe) complexes. Chem. Asian J..

[18] Weisheit T., Petzold H., Görls H., Mlostoń G., Weigand W. (2009). Reaction of 3,3,5,5-tetraphenyl-1,2,4-trithiolane with Pt^0^(bisphosphine)(*η*^2^-nbe) complexes bearing bridged bisphosphine ligands with various bite angles. Eur. J. Inorg. Chem..

[19] Mlostoń G., Romański J., Weigand W., Heimgartner H. (2019). Organic and coordination chemistry of 1,2,4-trithiolanes. Eur. J. Org. Chem..

[20] Petzold H., Görls H., Weigand W. (2007). A simple and efficient synthesis of bisphosphine platinum(0) complexes with various P–Pt–P angles. J. Organomet. Chem..

[21] COLLECT (1998). Data Collection Software.

[22] Otwinowski Z., Minor W., Carter C.W., Sweet R.M. (1997). Processing of X-Ray Diffraction Data Collected in Oscillation Mod. Methods in Enzymology Macromolecular Crystallography, Part A.

[23] Sheldrick G.M. (2002). SADABS 2.10.

[24] Sheldrick G.M. (2008). A short history of SHELX. Acta Cryst..

[25] Sheldrick G.M. (2015). Crystal structure refinement with SHELXL. Acta Cryst..

[26] Spek A.L. (2015). PLATON SQUEEZE: A tool for the calculation of the disordered solvent contribution to the calculated structure factors. Acta Cryst..

[27] Macrae C.F., Edgington P.R., McCabe P., Pidcock E., Shields G.P., Taylor R., Towler M., van de Streek J. (2006). Mercury: Visualization and analysis of crystal structures. J. Appl. Cryst..

[28] Becke A.D. (1998). Density-functional exchange-energy approximation with correct asymptotic behavior. Phys. Rev. A.

[29] Perdew J.P. (1986). Density-functional approximation for the correlation energy of the inhomogeneous electron gas. Phys. Rev. B.

[30] Grimme S., Antony J., Ehrlich S., Krieg H. (2010). A consistent and accurate ab initio parametrization of density functional dispersion correction (DFT-D) for the 94 elements H-Pu. J. Chem. Phys..

[31] Grimme S., Ehrlich S., Goerigk L. (2011). Effect of the damping function in dispersion corrected density functional theory. J. Comput. Chem..

[32] Eichkorn K., Treutler O., Öhm H., Häser M., Ahlrichs R. (1995). Auxiliary basis sets to approximate Coulomb potentials. Chem. Phys. Lett..

[33] Eichkorn K., Weigend F., Treutler O., Ahlrichs R. (1997). Auxiliary basis sets for main row atoms and transition metals and their use to approximate Coulomb potentials. Theor. Chem. Acc..

[34] Weigend F., Ahlrichs R. (2005). Balanced basis sets of split valence, triple zeta valence and quadruple zeta valence quality for H to Rn: Design and assessment of accuracy. Phys. Chem. Chem. Phys..

[35] Weigend F. (2006). Accurate Coulomb-fitting basis sets for H to Rn. Phys. Chem. Chem. Phys..

[36] Ahlrichs R., Armbruster M.K., Bachorz R.A., Bahmann H., Bär M., Baron H.-P., Bauernschmitt R., Bischoff F.A., Böcker S., Burow A.M. TURBOMOLE 7.2, University of Karlsruhe and Forschungszentrum Karlsruhe GmbH, 1989–2007, TURBOMOLE GmbH, since 2007, 2017. http://www.turbomole.com.

[37] Frisch M.J., Trucks G.W., Schlegel H.B., Scuseria G.E., Robb M.A., Cheeseman J.R., Scalmani G., Barone V., Mennucci B., Petersson G.A. (2013). Gaussian 09 D.01.

[38] Reed A.E., Curtiss L.A., Weinhold F. (1988). Intermolecular interactions from a natural bond orbital, donor-acceptor viewpoint. Chem. Rev..

[39] Bader R.F.W. (1990). Atoms in Molecules: A Quantum Theory.

[40] Glendening E.D., Reed A.E., Carpenter J.E., Weinhold F. (1993). NBO 3.1.

[41] Lu T., Chen F. (2012). Multiwfn: A multifunctional wavefunction analyzer. J. Comput. Chem..

[42] Lu T., Chen F. (2011). Calculation of molecular orbital composition. Acta Chim. Sin..

[43] Keith T.A. (2014). AIMAll 14.11.23.

[44] Becke A.D. (1993). Density-functional thermochemistry. III. The role of exact exchange. J. Chem. Phys..

[45] Vosko S.H., Wilk L., Nusair M. (1980). Accurate spin-dependent electron liquid correlation energies for local spin density calculations: A critical analysis. Can. J. Phys..

[46] Lee C., Yang W., Parr R.G. (1988). Development of the Colle-Salvetti correlation-energy formula into a functional of the electron density. Phys. Rev. B.

[47] Weisheit T., Petzold H., Görls H., Mlostoń G., Weigand W. (2010). Reactions of 1,2,4-trithiolanes and their 4-*S*-oxides with bisphosphane platinum(0) complexes. Eur. J. Inorg. Chem..

[48] Gorton J.E., Lentzner H.L., Watts W.E. (1971). Bridged ferrocenes—VIII: Polarographic half-wave potentials of ferrocenophanes and related compounds. Tetrahedron.

[49] Brown G.M., Meyer T.J., Cowan D.O., LeVanda C., Kaufman F., Roling P.V., Rausch M.D. (1975). Oxidation-state and electron-transfer properties of mixed-valence 1,1′-polyferrocene ions. Inorg. Chem..

[50] Camire N., Mueller-Westerhoff U.T., Geiger W.E. (2001). Improved electrochemistry of multi-ferrocenyl compounds: Investigation of biferrocene, terferrocene, bis(fulvalene)diiron and diferrocenylethane in dichloromethane using [NBu_4_][B(C_6_F_5_)_4_] as supporting electrolyte. J. Organomet. Chem..

[51] Ferguson G., Glidewell C., Opromolla G., Zakaria C.M., Zanello P. (1996). The redox behaviour of some bis-ferrocenyl compounds: Crystal and molecular structures of diferrocenylmethane and diferrocenylmethanol. J. Organomet. Chem..

[52] Hedberg F.L., Rosenberg H. (1969). Synthesis of 1,1′-biferrocenylene. J. Am. Chem. Soc..

[53] Zanello P., Opromolla G., Herberhold M., Brendel H.D. (1994). Redox behaviour of ferrocene derivatives VII. Chalcogen-bridged tri- and tetra-nuclear ferrocenes. J. Organomet. Chem..

[54] Robin M.B., Day P. (1967). Mixed valence chemistry—A survey and classification. Adv. Inorg. Chem. Radiochem..

[55] Xie R.J., Han L.M., Zhu N., Hong H.L., Suo Q.L., Ke C.L. (2013). Synthesis, crystal structure and electrochemistry of carbon-bridged diferrocenyl compounds. Asian J. Chem..

[56] Wiberg K.B. (1968). Application of the Pople-Santry-Segal CNDO method to the cyclopropylcarbinyl and cyclobutylcation and to bicyclobutane. Tetrahedron.

[57] Angyan J.G., Loos M., Mayer I. (1994). Covalent bond orders and atomic valence indices in the topological theory of atoms in molecules. J. Phys. Chem..

[58] Macchi P., Sironi A. (2003). Chemical bonding in transition metal carbonyl clusters: Complementary analysis of theoretical and experimental electron densities. Coord. Chem. Rev..

[59] Baryshnikov G.V., Minaev B.F., Minaeva V.A., Podgornaya A.T., Ågren H. (2012). Application of Bader’s atoms in molecules theory to the description of coordination bonds in the complex compounds of Ca^2+^ and Mg^2+^ with methylidene rhodanine and its anion. Russ. J. Gen. Chem..

[60] Alexiou A.D.P., Decandio C.C., da NAlmeida S., Ferreira M.J.P., Romoff P., Rocha R.C. (2017). Metal-ligand coordination and antiradical activity of a trichromium(III) complex with the flavonoid naringenin. J. Coord. Chem..

[61] Bader R.F.W., Essén H. (1984). The characterization of atomic interactions. J. Chem. Phys..

[62] Cremer D., Kraka E. (1984). Chemical bonds without bonding electron density—Does the difference electron-density analysis suffice for a description of the chemical bond?. Angew. Chem. Int. Ed..

[63] Espinosa E., Alkorta I., Elguero J., Molins E. (2002). From weak to strong interactions: A comprehensive analysis of the topological and energetic properties of the electron density distribution involving X–H···F–Y systems. J. Chem. Phys..

[64] Shahi A., Arunan E. (2014). Hydrogen bonding, halogen bonding and lithium bonding: An atoms in molecules and natural bond orbital perspective towards conservation of total bond order, inter- and intra-molecular bonding. Phys. Chem. Chem. Phys..

[65] Caldwell L.M., Hill A.F., Stranger R., Terrett R.N.L., von Nessi K.M., Ward J.S., Willis A.C. (2015). Thioxoethenylidene (CCS) as a Bridging Ligand. Organometallics.

[66] Heinze K., Lang H., Heinze K., Lang H. (2013). Ferrocene—Beauty and Functions. Organometallics.

[67] Dumas P., Girault J.-P., Guerin P. (1980). Polymerization of methylthiirane initiated by platinum(II)- and palladium(II) complexes. Polym. Bull..

[68] Schenk W.A. (2011). The coordination chemistry of small sulfur-containing molecules: A personal perspective. J. Chem. Soc. Dalton Trans..

[69] Lubitz W., Ogata H., Rüdiger O., Reijerse E. (2014). Hydrogenases. Chem. Rev..

[70] Li Y., Rauchfuss T.B. (2016). Synthesis of Diiron(I) Dithiolato Carbonyl Complexes. Chem. Rev..

[71] Daraosheh A.Q., Görls H., El-Khateeb M., Mloston G., Weigand W. (2011). Reactions of selected aromatic thioketones with dodecarbonyltriiron. Eur. J. Inorg. Chem..

[72] Daraosheh A.Q., Apfel U.-P., Görls H., Friebe C., Schubert U.S., El-Khateeb M., Mloston G., Weigand W. (2012). New approach to [FeFe]-hydrogenase models using aromatic thioketones. Eur. J. Inorg. Chem..

