# Supplementary material for: Diferrocenyl Thioketone: Reactions with (Bisphosphane)Pt(0) Complexes—Electrochemical and Computational Studies
Source: Materials (Basel). 2019 Sep 3;12(17):2832. doi: 10.3390/ma12172832 (PMC6747965; doi:10.3390/ma12172832)
Supplement: Supplementary file 1 [file materials-12-02832-s001.pdf]

materials

MDPI

Supplementary material

Diferrocenyl Thioketone: Reactions with (Bisphosphane)Pt(0) Complexes—Electrochemical and Computational Studies

Sebastian Gröber [1,2], Piotr Matczak [3], Sławomir Domagała [4], Thomas Weisheit [2], Helmar Görls [1], Annika Düver [1], Grzegorz Mlostoń [5,*] and Wolfgang Weigand [1,*]

1 Institute for Inorganic and Analytical Chemistry, Friedrich-Schiller University Jena, Humboldstrasse 8, 07743 Jena, Germany
2 Clinic of Nuclear Medicine, University Hospital Jena, Am Klinikum 1, 07747 Jena, Germany.
3 Department of Physical Chemistry, Faculty of Chemistry, University of Łódź, Pomorska 163/165, 90236 Lodz, Poland.
4 Department of Inorganic and Analytical Chemistry, Faculty of Chemistry, University of Łódź, Tamka 12, 91403 Łódź, Poland.
5 Department of Organic and Applied Chemistry, Faculty of Chemistry, University of Łódź, Tamka 12, 91403 Łódź, Poland
* Correspondence: grzegorz.mloston@chemia.uni.lodz.pl (G.M.); wolfgang.weigand@uni-jena.de (W.W.); Tel.: +49-3641-948160 (W.W.); Tel.: +48-42-6355761 (G.M.)

S1. Further Computational Details

The geometry of complex **3c** in its ground electronic state was optimized in the gas phase with a density functional theory (DFT) method. A combination of Becke's 1988 exchange (B) [1] and Perdew's 1986 correlation (P) [2] was selected. The resulting exchange-correlation functional was further improved by incorporating the respective Grimme's D3 dispersion correction [3] with Becke–Johnson damping [4]. This dispersion-corrected functional is denoted in the present work by the abbreviation BP-D. The def2-TZVP basis set [5] was assigned to the atoms of the complex. This basis set replaced 60 core electrons of Pt by a relativistic effective core potential [6]. The resolution-of-the-identity (RI-J) technique [7–9] was used to calculate Coulomb integrals in a cost-effective way. The "m4" grid size was set for the quadrature of exchange-correlation potential. The XRD geometry of complex **3c** (see Table S1) was the starting point for the optimization. No symmetry constraints were imposed and the optimized geometry fulfilled tight convergence criteria (thresholds < 10–5 au). The calculation of harmonic vibrational frequencies in the complex was performed analytically at the BP-D/def2-TZVP level. The lack of any imaginary frequencies confirmed that the optimized geometry corresponded to a local minimum on the potential energy surface. It is worth noting that BP-D/def2-TZVP predicted a singlet spin multiplicity for the ground electronic state of both the initial XRD and optimized geometries of the complex. For **3c** in its BP-D/def2-TZVP-optimized geometry, a single-point energy calculation was carried out at the BP-D/def2-TZVPP [1–5] level and without the RI-J technique in order to improve the accuracy of the molecular wave function. The augmentation of a basis set from def2-TZVP to def2-TZVPP increased the number of primitive Gaussians from 3429 to 4284. The molecular wave function generated by the BP-D/def2-TZVPP single-point energy calculation was further analyzed by the NBO [10] and QTAIM [11] methods. The "1st-order Promega" algorithm was used as a basic integration method in the QTAIM calculations.

Optimization and vibrational frequency calculations were also performed for the separate thioketone and (dppf)Pt fragments constituting complex **3c** and for a molecule of *tert*-butylthiol (this molecule was included for purposes of comparison). The starting structures of the two fragments for the optimizations were cut out from the XRD structure of complex **3c**. The computational

methodology applied to studying the two fragments and the *tert*-butylthiol molecule was the same as that used for complex **3c**.

Table S1. Selected bond lengths (in Å) and angles (in °) measured for complexes **3a**–**c** using XRD methods.

	**3a**	**3b**	**3c**
P(1)–Pt	2.2384(8)	2.2301(7)	2.2550(10)
P(2)–Pt	2.2719(8)	2.2507(7)	2.2877(9)
S(1)–Pt	2.3014(8)	2.2990(7)	2.2937(9)
C(1)–Pt	2.155(3)	2.154(3)	2.157(3)
C(1)–S(1)	1.791(3)	1.789(3)	1.784(4)
C(1)–C(2)	1.493(4)	1.485(4)	1.494(5)
C(1)–C(12)	1.494(4)	1.496(4)	1.494(5)
Fe(1)–Fe(2) (intra/intermolecular)	5.668/5.375	5.226/9.608	5.241/5.680
P(1)–Pt–P(2)	86.79(3)	88.17(3)	103.28(3)
P(2)–Pt–S(1)	112.08(3)	115.11(3)	104.31(3)
P(1)–Pt–C(1)	161.13(3)	110.05(8)	105.17(10)
C(1)–Pt–S(1)	47.25(8)	47.24(8)	47.15(10)
Pt–S(1)–C(1)	62.07(10)	62.12(9)	62.39(11)
S(1)–C(1)–Pt	70.68(11)	70.64(10)	70.47(12)
C(2)–C(1)–C(12)	111.5(3)	118.5(3)	116.6(3)
Sum of angles around Pt center	360,00	360,57	359,91

Table S2. Calculated NBO and QTAIM indicators of bond order for the bonds around the Pt center in complex **3c**. For some of the bonds, their order indices calculated for the isolated **1a** and (dppf)Pt are presented in parentheses. These results were obtained from the B3LYP/def2-TZVPP wave functions of BP-D/def2-TZVP-optimized structures.

**Bond**	**WBI**	**DI**
Pt–P(1)	0.640 (0.723)	1.040 (1.164)
Pt–P(2)	0.619 (0.723)	0.982 (1.165)
Pt–S(1)	0.682	0.912
Pt–C(1)	0.433	0.713
C(1)–S(1)	1.155 (1.688)	1.164 (1.718)

Table S3. Calculated QTAIM parameters of bond critical points for the bonds around the Pt center in complex **3c**. For some of the bonds, their QTAIM parameters calculated for the isolated **1a** and (dppf)Pt are presented in parentheses. These results were obtained from the B3LYP/def2-TZVPP wave functions of BP-D/def2-TZVP-optimized structures.

**Bond**	**ρ (au)**	**$\nabla^2\rho$ (au)**	**H (au)**	**$-V/G$**	**$-\lambda_1/\lambda_3$**
Pt–P(1)	0.1241	0.0912	−0.0644	1.7395	0.3514
	(0.1284)	(0.0748)	(−0.0710)	(1.7923)	(0.3814)
Pt–P(2)	0.1157	0.1074	−0.0558	1.6759	0.3242
	(0.1285)	(0.0748)	(−0.0711)	(1.7924)	(0.3814)
Pt–S(1)	0.1008	0.1461	−0.0400	1.5229	0.2915
Pt–C(1)	0.1111	0.1195	−0.0464	1.6089	0.3688
C(1)–S(1)	0.1863	−0.2861	−0.1392	3.0575	1.2671
	(0.2281)	(−0.3207)	(−0.2708)	(2.4206)	(1.4192)

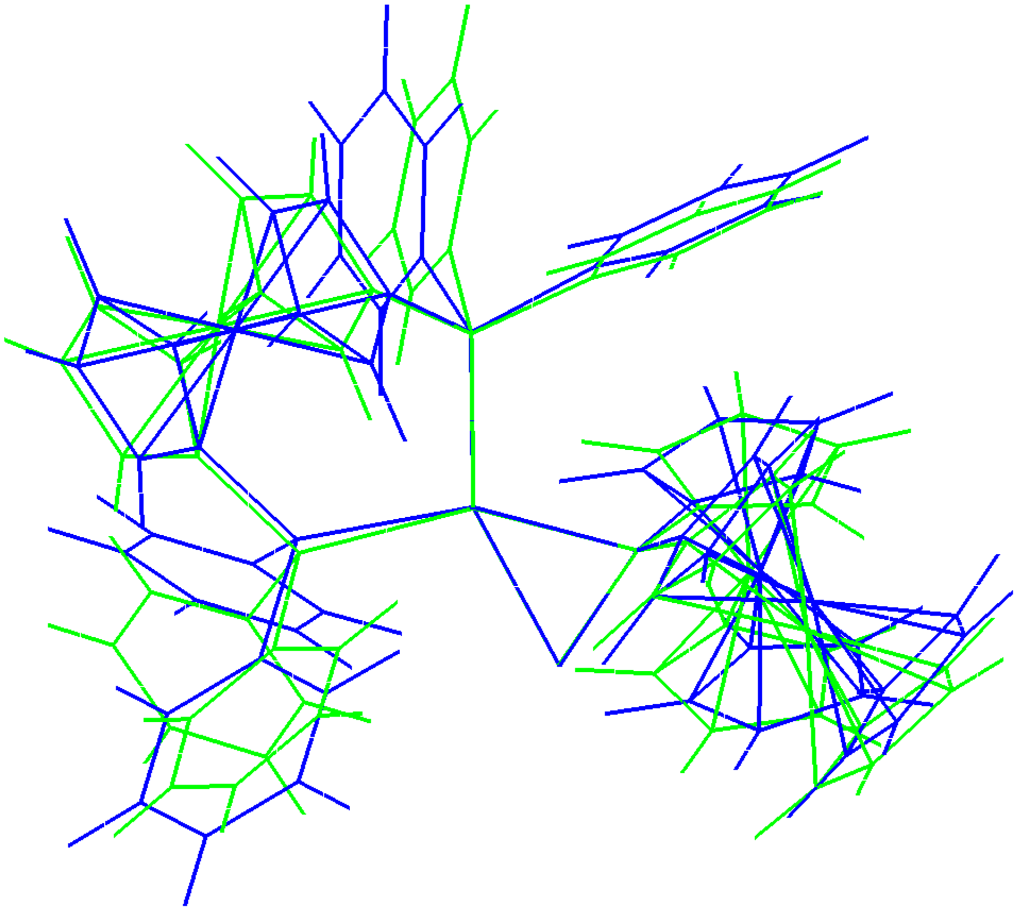

Figure S1. Superposition of the calculated (blue) and experimental (green) structures of complex **3c**.

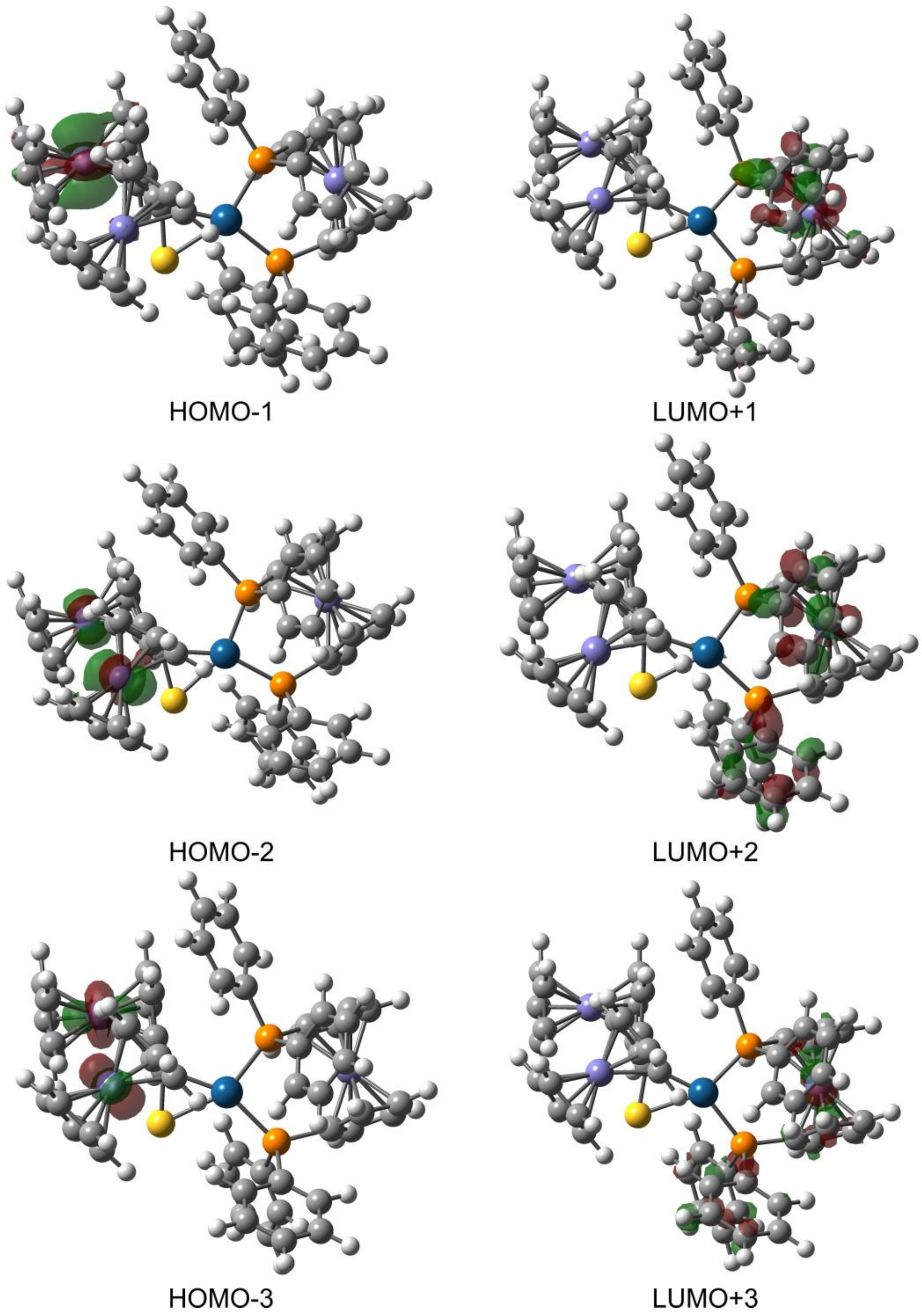

Figure S2. Contours of several molecular orbitals below and above the frontier molecular orbitals for complex **3c**. Their parts possessing either positive or negative sign are colored red and green, respectively. The contours are plotted with an isovalue of 0.05 au. The orbitals were obtained from the BP-D/def2-TZVPP wave function of the complex.

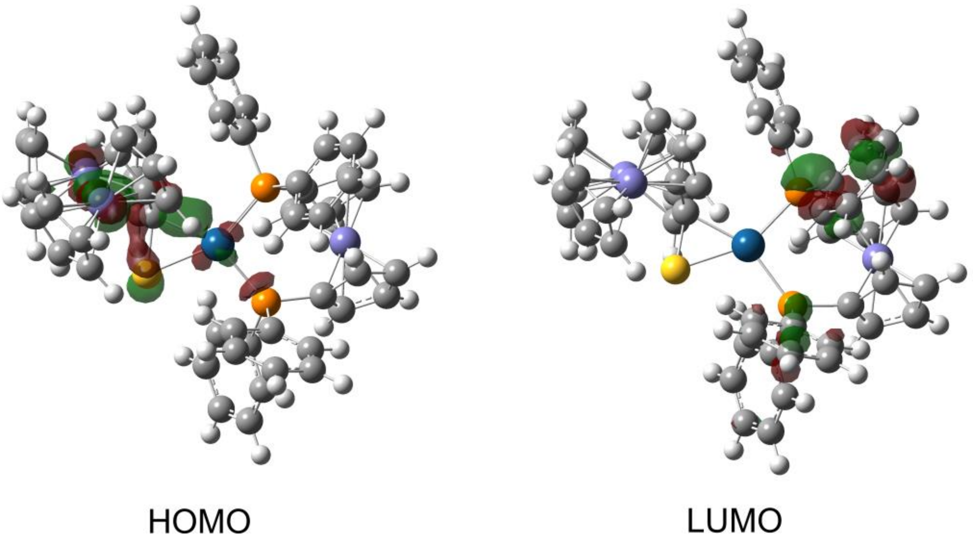

Figure S3. Contours of the highest occupied molecular orbital and lowest unoccupied molecular orbital for complex **3c**. Their parts possessing either positive or negative sign are colored red and green, respectively. The contours are plotted with an isovalue of 0.05 au. The orbitals were obtained from the B3LYP/def2-TZVPP wave function of the BP-D/def2-TZVP-optimized complex.

S2. Further Electrochemical Details

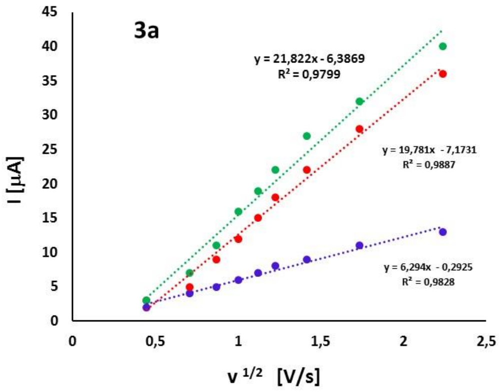

(a)

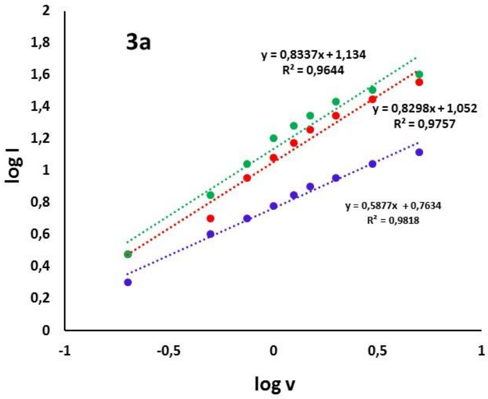

(b)

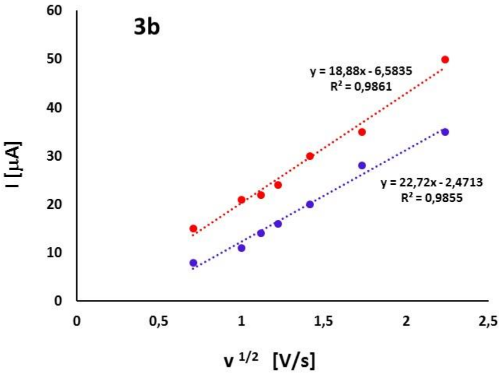

(c)

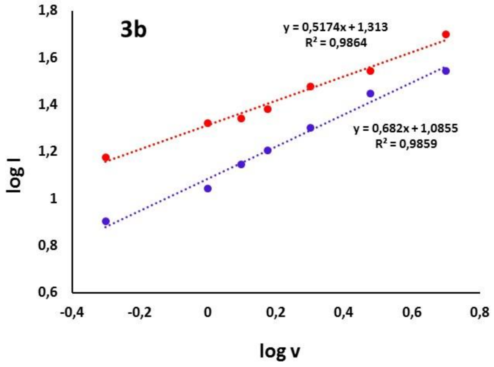

(d)

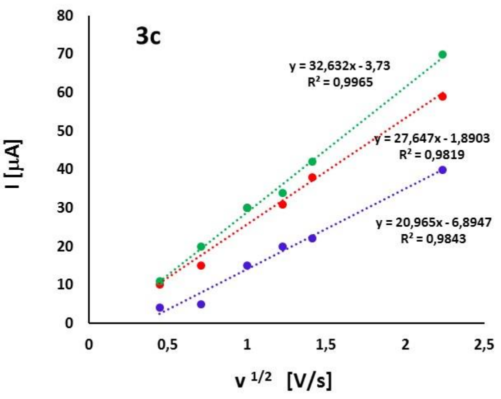

(e)

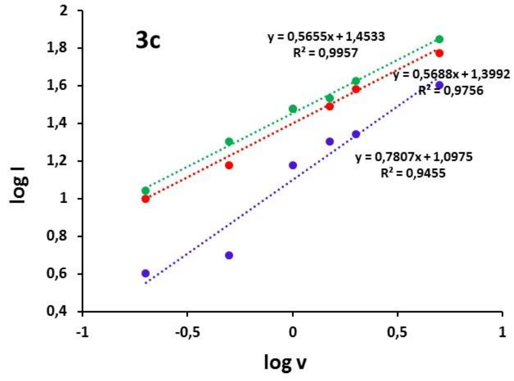

(f)

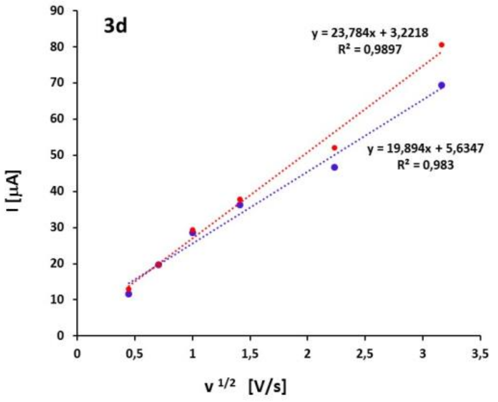

(g)

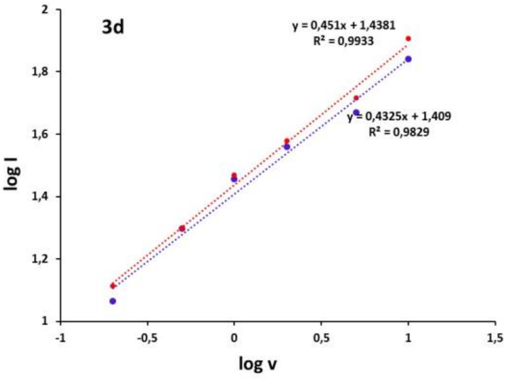

(h)

Figure S4. Dependence of the peak currents (*I*) from the square root of scan rate ($v^{1/2}$) and logarithm of peak currents (*log I*) versus logarithm of scan rates (*log v*) for complexes **3a–d** (1.0 mmol/L) in CH_2Cl_2—[*n*Bu_4N][BF_4] (0.1 m/L).

S3. Further Crystallographic Data

Table S4. Crystal data and refinement details for the X-ray structure determinations.

Compound	3a	3b	3c
formula	$C_{58}H_{50}Fe_2P_2PtS$	$C_{56}H_{46}Cl_2Fe_2P_2PtS$	$C_{55}H_{46}Fe_3P_2PtS$ [*]
fw (g·mol^{-1})	1147.77	1190.62	1163.56 [*]
T/°C	−140 (2)	−140 (2)	−140 (2)
crystal system	monoclinic	monoclinic	triclinic
space group	P 2_1/c	P 2_1/n	P$\bar{1}$
a/ Å	9.2351 (2)	22.1669 (3)	11.0264 (5)
b/ Å	33.6468 (7)	9.6079 (1)	12.7663 (6)
c/ Å	15.3738 (3)	23.6359 (3)	17.8090 (7)
α/°	90	90	83.222 (2)
β/°	101.479 (1)	110.337 (1)	79.406 (2)
γ/°	90	90	76.464 (2)
V/Å^3	4681.57 (17)	4720.12 (10)	2388.34 (18)
Z	4	4	2
ρ (g·cm^{-3})	1.628	1.667	1.618 [*]
μ (cm^{-1})	37.45	38.27	39.6 [*]
measured data	33532	33475	11623
data with I > 2σ(I)	9626	9802	9835
unique data (R_{int})	10592/0.0408	10719/0.0362	10453/0.0296
wR_2 (all data, on F^2)$^{a)}$	0.0603	0.0564	0.0719

R_1 ($I > 2\sigma(I)$) [a]	0.0300	0.0269	0.0308
S [b]	1.111	1.074	1.043
Res. dens./e·Å^{-3}	0.734/−0.519	0.612/−0.686	0.978/−1.911
absorpt method	multi-scan	multi-scan	multi-scan
absorpt corr $T_{min/max}$	0.6690/0.7456	0.6872/0.7456	0.5931/0.7456
CCDC No.	1922306	1922307	1922308

[*] derived parameters do not contain the contribution of the disordered solvent. [a] Definition of the R indices: $R_1 = (\Sigma || F_o| - |F_c||)/\Sigma |F_o|$; $wR_2 = \{\Sigma[w(F_o^2-F_c^2)^2]/\Sigma[w(F_o^2)^2]\}^{1/2}$ with $w^{-1} = \sigma^2(F_o^2) + (aP)^2 + bP$; $P = [2F_c^2 + Max(F_O^2]/3$; [b] $s = \{\Sigma[w(F_o^2 - F_c^2)^2]/(N_o - N_p)\}^{1/2}$.

S4. Cartesian Coordinates (in Å) for 3c, 1a and (dppf)Pt-Fragment

Table S5. Cartesian coordinates (in Å) for complex **3c** optimized at the BP-D/def2-TZVP level of theory.[1.]

Atom	X (in Å)	Y (in Å)	Z (in Å)
Pt	6.349591	7.331747	12.635139
Fe	5.394941	11.959588	11.076489
Fe	4.572142	10.313061	15.861404
Fe	9.211537	4.795696	10.886714
S	4.228175	8.284804	12.658337
P	8.579137	7.428944	12.887853
P	6.103759	5.10597	12.196501
C	5.639419	9.341508	12.955411
C	6.0997	10.178027	11.827487
C	7.125248	11.1913	11.84091
H	7.663046	11.536407	12.71554
C	7.33142	11.652654	10.502553
H	8.035278	12.422828	10.199914
C	6.432232	10.939516	9.644807
H	6.320845	11.080356	8.573322
C	5.671561	10.041229	10.457821
H	4.879636	9.375463	10.131817
C	3.883087	12.524139	12.325295
H	3.627835	11.988271	13.232808
C	4.858458	13.570475	12.215827
H	5.434133	13.997591	13.032378
C	4.95362	13.947054	10.835152
H	5.617959	14.700948	10.42293
C	4.036738	13.132889	10.091871
H	3.888222	13.158794	9.016185
C	3.376331	12.252788	11.013335
H	2.649135	11.487417	10.759397
C	5.886756	9.815498	14.342529
C	6.040422	8.961508	15.492952
H	5.919423	7.882709	15.476099
C	6.377968	9.768469	16.626016
H	6.547481	9.409772	17.637284
C	6.408171	11.136479	16.196015
H	6.603598	12.001257	16.824095
C	6.096886	11.165679	14.79782
H	6.020612	12.049763	14.176295
C	2.679157	10.295549	15.080108

H	2.452453	10.172386	14.025482
C	2.849043	9.227106	16.017531
H	2.772842	8.169867	15.784667
C	3.19673	9.801358	17.285041
H	3.418741	9.253023	18.196102
C	3.245759	11.225894	17.12748
H	3.505793	11.945242	17.89869
C	2.923998	11.531283	15.764197
H	2.890112	12.524642	15.326264
C	9.474838	6.707149	11.494914
C	8.910816	6.677108	10.167143
H	7.942809	7.093251	9.902663
C	9.818226	5.978397	9.313078
H	9.660156	5.758434	8.261427
C	10.94554	5.572991	10.097707
H	11.791038	4.988803	9.745403
C	10.740449	6.02051	11.441206
H	11.396181	5.832652	12.284717
C	7.482901	4.050187	11.665509
C	7.692644	3.537769	10.335655
H	7.021876	3.687134	9.495815
C	8.939441	2.838153	10.316545
H	9.390092	2.366402	9.448179
C	9.508324	2.906082	11.628404
H	10.466402	2.494978	11.932945
C	8.62073	3.657097	12.461951
H	8.778368	3.90658	13.505774
C	9.207463	6.558533	14.371076
C	10.555096	6.634089	14.753595
H	11.243991	7.264396	14.189594
C	11.00837	5.928476	15.868448
H	12.058351	5.988507	16.15817
C	10.117504	5.154857	16.620253
H	10.474801	4.606867	17.493099
C	8.768142	5.102078	16.26333
H	8.062315	4.516052	16.853003
C	8.315774	5.805534	15.14572
H	7.263141	5.777954	14.861321
C	9.353244	9.070405	13.069927
C	9.204066	9.75827	14.284615
H	8.657571	9.299527	15.108442
C	9.735921	11.037486	14.43377
H	9.604114	11.565493	15.378568
C	10.420685	11.642668	13.374663
H	10.834619	12.644868	13.492615
C	10.566176	10.962892	12.163418
H	11.091399	11.432801	11.330989
C	10.031917	9.682484	12.008098
H	10.137986	9.159104	11.057962
C	4.900808	4.79061	10.853927
C	4.666105	5.803189	9.914869
H	5.153619	6.770349	10.048699

C	3.79291	5.581088	8.848285
H	3.612103	6.374961	8.122542
C	3.140473	4.352632	8.722569
H	2.450151	4.182624	7.895273
C	3.36231	3.343785	9.665798
H	2.844413	2.387894	9.576406
C	4.242071	3.559531	10.726446
H	4.41136	2.773523	11.463763
C	5.423705	4.202231	13.637365
C	5.767391	2.877638	13.94275
H	6.453948	2.331491	13.295772
C	5.246133	2.261134	15.082722
H	5.523966	1.232158	15.315521
C	4.375136	2.959324	15.922326
H	3.973303	2.477956	16.814873
C	4.023393	4.278087	15.617578
H	3.344282	4.828503	16.269992
C	4.546894	4.900478	14.485081
H	4.2944	5.938817	14.253057

[1] The total energy amounts to -7117.47021285869 hartree.

Table S6. Cartesian coordinates (in Å) for thioketone **1a** optimized at the BP-D/def2-TZVP level of theory.[1.]

Atom	X (in Å)	Y (in Å)	Z (in Å)
Fe	5.069636	11.854642	11.338941
Fe	4.855314	10.231233	16.141428
S	5.080581	7.933517	13.061739
C	5.758559	9.445478	13.227784
C	6.097861	10.236068	12.05056
C	6.949324	11.409817	11.983096
H	7.482839	11.852581	12.816787
C	6.995326	11.848988	10.6258
H	7.54185	12.712035	10.256518
C	6.169273	10.976436	9.844281
H	5.972978	11.070777	8.780335
C	5.614366	9.991223	10.71246
H	4.935898	9.187387	10.448133
C	3.53303	12.249569	12.635874
H	3.396423	11.71913	13.571884
C	4.339618	13.418549	12.449065
H	4.874134	13.960082	13.224124
C	4.329487	13.74359	11.052886
H	4.862083	14.56581	10.584424
C	3.515363	12.774134	10.379607
H	3.327938	12.731224	9.310848
C	3.024188	11.84855	11.359207
H	2.40407	10.978927	11.165126
C	6.114539	9.977097	14.546982
C	6.506327	9.159088	15.673786
H	6.570559	8.076294	15.64424
C	6.788187	10.018941	16.778335
H	7.097907	9.698973	17.768875

C	6.543927	11.370571	16.368484
H	6.629978	12.253213	16.995855
C	6.128645	11.350902	15.000316
H	5.838602	12.208934	14.405508
C	2.959456	9.822396	15.490857
H	2.712214	9.618864	14.452579
C	3.392264	8.840595	16.438973
H	3.51522	7.781491	16.236534
C	3.695143	9.518278	17.66625
H	4.074714	9.057061	18.573228
C	3.452073	10.917969	17.472882
H	3.6106	11.701475	18.207997
C	2.993478	11.105117	16.1283
H	2.7357	12.057724	15.674673

[1] The total energy amounts to -3737.75343192882 hartree. The starting geometry for the optimization was cut out from the XRD structure of **3c**.

Table S7. Cartesian coordinates (in Å) for (dppf)Pt-fragment optimized at the BP-D/def2-TZVP level of theory.[1].

Atom	X (in Å)	Y (in Å)	Z (in Å)
Pt	6.732068	6.635746	13.563883
Fe	8.985858	4.564078	11.385604
P	8.795975	7.399264	13.332294
P	5.809973	4.822257	12.694103
C	9.40505	6.514069	11.861078
C	8.66869	6.43294	10.625091
H	7.689908	6.867847	10.453944
C	9.426069	5.650388	9.699867
H	9.120498	5.385165	8.691957
C	10.628854	5.230969	10.355499
H	11.396977	4.589981	9.931742
C	10.618623	5.75991	11.686615
H	11.373497	5.600361	12.450023
C	7.248081	3.825989	12.185378
C	7.527527	3.208195	10.915658
H	6.89009	3.260218	10.038744
C	8.80046	2.557524	11.005601
H	9.303448	2.030172	10.199793
C	9.316862	2.768683	12.325108
H	10.279219	2.43015	12.697687
C	8.368274	3.553918	13.050094
H	8.47445	3.920583	14.0653
C	10.009822	6.894645	14.612126
C	11.332345	7.360403	14.616133
H	11.654064	8.084194	13.86573
C	12.232471	6.908582	15.582825
H	13.259598	7.276513	15.581542
C	11.818482	5.991272	16.553487
H	12.523017	5.641887	17.309476
C	10.497551	5.534906	16.562743
H	10.166333	4.831116	17.327632
C	9.596123	5.989128	15.598883

H	8.552216	5.664193	15.597761
C	9.311317	9.124544	12.979732
C	8.860532	10.124564	13.855317
H	8.213392	9.84802	14.690804
C	9.225556	11.454681	13.655713
H	8.874444	12.223815	14.345084
C	10.028587	11.8044	12.564521
H	10.305109	12.84681	12.40136
C	10.466203	10.816967	11.679527
H	11.087095	11.086136	10.823611
C	10.113196	9.480597	11.886594
H	10.458664	8.710716	11.195925
C	4.910887	5.032216	11.108568
C	4.937975	6.29054	10.491651
H	5.445913	7.109312	11.008403
C	4.314247	6.485723	9.258446
H	4.339533	7.468855	8.786341
C	3.648897	5.425441	8.636914
H	3.155888	5.577463	7.675778
C	3.602591	4.172596	9.255981
H	3.072435	3.346779	8.779186
C	4.229546	3.975697	10.487637
H	4.183877	3.000234	10.974319
C	4.77251	3.580043	13.558399
C	5.070659	2.210612	13.582692
H	5.960056	1.841712	13.070804
C	4.23439	1.321136	14.263018
H	4.476103	0.257274	14.280738
C	3.092912	1.790269	14.916329
H	2.441491	1.094678	15.446912
C	2.792351	3.156699	14.897636
H	1.906251	3.528851	15.413778
C	3.633074	4.046868	14.23192
H	3.418219	5.117884	14.234495

[1] The total energy amounts to -3379.61267916211 hartree. The starting geometry for the optimization was cut out from the XRD structure of **3c**.

References

1. Becke, A.D. Density-functional exchange-energy approximation with correct asymptotic behavior. *Phys. Rev. A* **1988**, *38*, 3098–3100.
2. Perdew, J.P. Density-functional approximation for the correlation energy of the inhomogeneous electron gas. *Phys. Rev. B* **1986**, *33*, 8822–8824.
3. Grimme, S.; Antony, J.; Ehrlich, S.; Krieg, H. A consistent and accurate ab initio parametrization of density functional dispersion correction (DFT-D) for the 94 elements H-Pu. *J. Chem. Phys.* **2010**, *132*, 154104.
4. Grimme, S.; Ehrlich, S.; Goerigk, L. Effect of the damping function in dispersion corrected density functional theory. *J. Comput. Chem.* **2011**, *32*, 1456–1465.
5. Weigend, F.; Ahlrichs, R. Balanced basis sets of split valence, triple zeta valence and quadruple zeta valence quality for H to Rn: design and assessment of accuracy. *Phys. Chem. Chem. Phys.* **2005**, *7*, 3297–3305.
6. Andrae, D.; Häußermann, U.; Dolg, M.; Stoll, H.; Preuß, H. Energy-adjusted ab initio pseudopotentials for the second and third row transition elements. *Theor. Chem. Acc.* **1990**, *77*, 123–141.
7. Eichkorn, K.; Treutler, O.; Öhm, H.; Häser, M.; Ahlrichs, R. Auxiliary basis sets to approximate Coulomb potentials. *Chem. Phys. Lett.* **1995**, *240*, 283–290.

8. Eichkorn, K.; Weigend, F.; Treutler, O.; Ahlrichs, R. Auxiliary basis sets for main row atoms and transition metals and their use to approximate Coulomb potentials. *Theor. Chem. Acc.* **1997**, *97*, 119–124.
9. Weigend, F. Accurate Coulomb-fitting basis sets for H to Rn. *Phys. Chem. Chem. Phys.* **2006**, *8*, 1057–1065.
10. Reed, A.E.; Curtiss, L.A.; Weinhold, F. Intermolecular interactions from a natural bond orbital, donor-acceptor viewpoint. *Chem. Rev.* **1988**, *88*, 899–926.
11. Bader, R.F.W. *Atoms in Molecules: A Quantum Theory*. Clarendon: Oxford, UK, 1990.

© 2019 by the authors. Submitted for possible open access publication under the terms and conditions of the Creative Commons Attribution (CC BY) license (http://creativecommons.org/licenses/by/4.0/).
